# A Novel Antimicrobial Metabolite Produced by *Paenibacillus apiarius* Isolated from Brackish Water of Lake Balkhash in Kazakhstan

**DOI:** 10.3390/microorganisms10081519

**Published:** 2022-07-27

**Authors:** Alexander Meene, Christiane Herzer, Rabea Schlüter, Bolatkhan Zayadan, Ruediger Pukall, Peter Schumann, Frieder Schauer, Tim Urich, Annett Mikolasch

**Affiliations:** 1 Institute of Microbiology, University Greifswald, Felix-Hausdorff-Straße 8, 17487 Greifswald, Germany; alex.meene@web.de (A.M.); christiane.herzer@gmail.com (C.H.); frieder.schauer@uni-greifswald.de (F.S.); tim.urich@uni-greifswald.de (T.U.); 2 Imaging Center of the Department of Biology, University Greifswald, Friedrich-Ludwig-Jahn-Str. 15, 17487 Greifswald, Germany; rabea.schlueter@uni-greifswald.de; 3 Department of Biotechnology, Al-Farabi Kazakh National University, 71, al-Farabi Ave, Almaty 050040, Kazakhstan; bolatkhan@kaznu.kz; 4 Leibniz Institute DSMZ—German Collection of Microorganisms and Cell Cultures, Inhoffenstrasse 7B, 38124 Braunschweig, Germany; rpu@dsmz.de (R.P.); schumann_peter@yahoo.de (P.S.)

**Keywords:** predation, bacterivory, lipopeptides, *Micrococcus luteus*, *Pseudomonas putida*, *Escherichia coli*, antimicrobial, antibiotic

## Abstract

Four aerobic bacteria with bacteriolytic capabilities were isolated from the brackish water site Strait Uzynaral of Lake Balkhash in Kazakhstan. The morphology and physiology of the bacterial isolates have subsequently been analyzed. Using matrix assisted laser desorption ionization-time of flight mass spectrum and partial 16S rRNA gene sequence analyses, three of the isolates have been identified as *Pseudomonas veronii* and one as *Paenibacillus apiarius*. We determined the capability of both species to lyse pre-grown cells of the Gram-negative strains *Pseudomonas putida* SBUG 24 and *Escherichia coli* SBUG 13 as well as the Gram-positive strains *Micrococcus luteus* SBUG 16 and *Arthrobacter citreus* SBUG 321 on solid media. The bacteriolysis process was analyzed by creating growth curves and electron micrographs of co-cultures with the bacteriolytic isolates and the lysis sensitive strain *Arthrobacter citreus* SBUG 321 in nutrient-poor liquid media. One metabolite of *Paenibacillus apiarius* was isolated and structurally characterized by various chemical structure determination methods. It is a novel antibiotic substance.

## 1. Introduction

Since the emergence of the first prokaryotes more than 3.5 billion years ago, microorganisms have developed highly complex interaction networks. In natural ecosystems, bacterial species normally closely interact with a variety of other species [1]. Synergy effects, or competition within a microenvironment or a specific ecological niche, led to the development of an enormous diversity of microbial interspecific interactions and nutrition types [2]. The ability of bacteria to digest bacterial cell walls and cells, referred to as bacteriolysis, is a specific property within saprotrophic and bacterivorous prokaryotes [3]. The term “bacterivorous prokaryotes” does not only include the parasitic and predatory BALOs (*Bdellovibrio* and like organisms) [4] and myxobacteria [5] but also intraguild predators. These bacteria share similar and often limited resources with their preys and usually produce antagonistic compounds. Intraguild predation (IGP), known as the competition of at least two non-related strains for nutrients, is proposed to be a very common phenomenon in nature. It is widespread in the prokaryotic world including lactic acid bacteria as well as members of the genus *Streptomyces* [6,7].

Many of the known bacterivorous prokaryotes show biological activity against a broad range of species and thus may have the potential to produce natural products that may be of use in biotechnological and medical applications. The mechanism of bacterivory often includes both the production of extracellular bacteriolytic enzymes [7,8,9] and the secretion of secondary metabolites [6,10,11].

Most of the studies performed to investigate antagonistic bacteria have applied standard screening procedures to search for secondary metabolites [12,13] and did not consider compounds that function only in a restricted ecological context [14]. In the past, field-approaches for studies of prokaryotic bacterivory in natural habitats were restricted by a lack of appropriate methodologies and by the fact that, in general, laboratory investigations are based on the use of pure cultures [8]. Today, molecular biology and genomic methods lead to a rapidly growing knowledge of ecologically relevant microbial processes, including those that involve unculturable bacterial species [3,15]. New basic knowledge on non-obligate predation (bacterivory) in nature is required to isolate novel bacterial producers of antibiotics and useful bacteriolytic extracellular enzymes which may be utilized to optimize biotechnological processes [16].

Though many soil-dwelling bacteria are already used industrially, microorganisms from aquatic habitats, in particular marine bacteria, are less explored. However, many of these also represent a promising source of novel bacteriolytic factors, and a huge reservoir of bioactive secondary metabolites, which might potentially find applications in green chemistry, pharmacy and biotechnology [17,18,19,20,21,22]. The aim of this study was to isolate and characterize facultative bacterivorous microorganisms from the brackish area of Lake Balkhash to recover new strains with extensive lysis capabilities that could also be cultured on nutrient agar for laboratory use.

Due to the ongoing desiccation of the Aral Sea, a result of different interferences in nature, Lake Balkhash in Kazakhstan became the biggest lake in Central Asia, at 17,000 km^2^ [23]. Similar to the Aral Sea, increased water consumptions along the main inflow River Ile, especially the dam construction at the Kapchagay reservoir in 1969, caused several environmental problems and a decrease in water level of around 1.5 m. The lake became of interest due to the desiccation problems and its uncommon limnological characteristics. Despite the streamed connection of the two lake parts at Strait Uzynaral, the south-western part is characterized by a lower salinity (<0.2%) than the eastern part (>0.4%) [24]. Several studies focusing on the water level problems [25,26] and resulting implications on the environment and biodiversity [23] were published in the past decade and helped to improve the understanding of the anthropogenic problems of this important ecosystem.

In order to learn more about microbial antagonism in the endangered habitat of Lake Balkhash and to find new antimicrobial agents and bacteriolytic enzymes, we have undertaken (i) the physiological screening and identification of selected bacteriolytic microorganisms isolated from the lake’s brackish water. (ii) We then analyzed the ability of these isolates to lyse different prey species, and (iii) we recorded growth curves of a co-culture containing one of the bacteriolytic isolates (either *Paenibacillus apiarius* SBUG 1947 (*P. apiarius*) or *Pseudomonas veronii* SBUG 1927 (*P. veronii*)) and the lysis sensitive strain *Arthrobacter citreus* SBUG 321 (*A. citreus*) in nutrient-poor liquid media. (iv) Electron microscopic studies were used to follow the bacteriolysis process of *P. apiarius* SBUG 1947 lysing *A. citreus* SBUG 321. (v) Finally, we have identified the molecular structure of the substance responsible for the antibacterial effects on different environmental and clinically relevant strains.

## 2. Materials and Methods

### 2.1. Sampling

**Figure 1:**
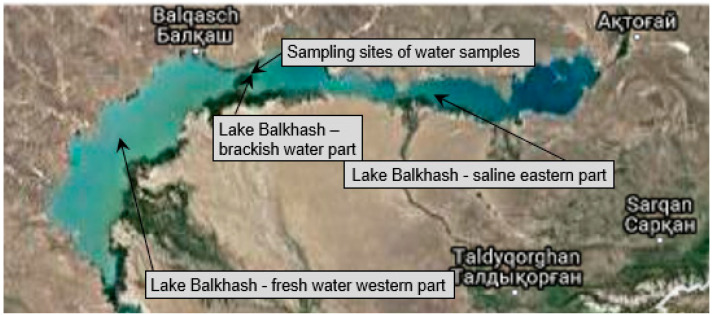
Sampling site of water samples A–E from Strait Uzynaral, Lake Balkhash (Kazakhstan).

Water samples were taken at Lake Balkhash in Kazakhstan, near Balkhash-City at Strait Uzynaral. Sampling sites and conditions are shown in Table 1 and Figure 1.

Strait Uzynaral separates the fresh water western part of the lake from the saline eastern part. It is a brackish water habitat with a salinity between 0.15% and 0.17% at the sampling site. As expected, the “eastern” samples had a higher salinity than the “western” samples, and the salinity was also higher 200 m in front of the reed belt than at a distance of only 100 m. The 10 mL water samples were filled into sterile falcon tubes and processed under sterile conditions on the same day according to Section 2.5.1.

### 2.2. Microorganisms

In the current work, living cells of *Arthrobacter citreus* SBUG 321 were used for isolation, because Christensen und Cook (1978) [27] reported that prey bacteria of the genus *Arthrobacter* are easily penetrated by bacteriolytic microorganisms. This finding was confirmed by the study of Brack et al. (2013) [3], in which bacteriolytic *Bacillus pumilus* and other bacteriolytic *Bacillus* species from the Baltic Sea were isolated using the genus *Arthrobacter* as a prey organism. The Gram-negative Proteobacteria *Pseudomonas putida* SBUG 24 and *Escherichia coli* SBUG 13 and the Gram-positive Actinobacterium *Micrococcus luteus* SBUG 16 were also used as living and autoclaved prey bacteria to determine the prey range. All strains that were used as prey bacteria originate from the culture collection of the Department of Applied Microbiology of the Institute of Microbiology at the University of Greifswald, Germany (SBUG).

### 2.3. Preparation of Bacteriolysis and Bacterivory Test Media

For the investigation of bacterivory behavior on living prey bacteria, double-layer agar plates were used. These plates consisted of a basal layer (bottom) and a thin top layer containing living cells of the prey *A. citreus*. For the basal layer, 1 L distilled water (dH_2_O) was mixed with 2% agar (*w*/*v*) (INVITROGEN, Karlsruhe, Germany) and 0.5% Bacto Peptone (*w*/*v*) (OTTO NORDWALD, Hamburg, Germany), adjusted to pH 6.5–7 and solved for 2 h at 100 °C. In order to ensure the identical volume of 12.5 mL per plate, the solved Bacto Peptone agar solution was bottled via Dispensette^®^ (Merck, Darmstadt, Germany) into glass test tubes, loosely sealed with a plug and autoclaved at 121 °C for 20 min. For later usage, test tubes with 4.5 mL of the Bacto Peptone agar were prepared for the top layer in the same way. Afterwards, the autoclaved test tubes with 12.5 mL agar were tempered at 60 °C and subsequently poured into an empty plate to create the thick basal layer. To prepare the top layer, living cells of *A. citreus* were incubated in 500 mL Erlenmeyer flasks with 100 mL sterile, pH adjusted 1.5% nutrient broth II (*w*/*v*) (SIFIN, Berlin, Germany) for 48 h at 30 °C and then harvested by centrifugation for 8 min at 3795× *g* at 4 °C. The supernatant was discarded, the pellet was weighted, and each 0.1 g of the weighted cell pellet was resuspended in 2.5 mL sterile, pH adjusted 0.5% Bacto Pepton (*w*/*v*) solution (e.g., 2 g cell pellet resuspended in 50 mL 0.5% Bacto Pepton solution) to get a cell suspension with a resulting cell concentration of 4% (*w*/*v*). Finally, 0.5 mL of this cell suspension was carefully mixed with the 4.5 mL of 0.5% Bacto Pepton agar solution in the previously bottled and autoclaved Bacto Pepton agar test tubes (pre-tempered at 50 °C to ensure the agar to be liquid and also avoid cell damage when adding the cell suspension) and poured on the solid basal layer. To ensure that the turbid lawn was formed by living prey bacteria, the plates were incubated for 48 h at 30 °C, and samples were streaked out to demonstrate viability. Thus, this lysis test medium contained living cells that were already cultivated to reach a high cell density—unlike the agar diffusion test for antibiotics, in which a small inoculum is used to detect growth inhibition. For the preparation of the bacteriolysis test media with autoclaved cells, a culture of *A. citreus* was incubated and harvested as set out above. The cell pellet was subsequently autoclaved for 20 min at 121 °C and then resuspended in 1.25 mL of 0.5% Bacto Pepton (*w*/*v*) each 0.1 g.

### 2.4. Preparation of the Co-Culture Media

In order to show significant bacterivorous activity, the isolated strains *P. veronii* SBUG 1927 and *P. apiarius* SBUG 1947 were tested separately in liquid co-culture media with the prey *A. citreus*. For this purpose, pure precultures of the corresponding isolate and prey strain were separately incubated in 500 mL Erlenmeyer flasks with 50 mL sterile, pH adjusted 1.5% nutrient broth II (*w*/*v*) (SIFIN, Berlin, Germany) for 24 h at 30 °C and then harvested by centrifugation (8 min at 3795× *g* at 4 °C). The cell pellets were resuspended in tenfold diluted 0.15% sterile, pH adjusted nutrient broth II (*w*/*v*) (DNB) to get an initial optical density at 500 nm (OD_500_) of 0.2 for the prey and 0.05 for the potentially bacteriolytic isolate. The cell counting of the optical density on agar plates into colony forming units (cfu) revealed cell counts for the prey organism of about between 1 × 10^8^ and 2 × 10^8^ cfu and for the isolate of about 5 × 10^4^ cfu. The cell counts for isolate to prey revealed a ratio of 1:5000, ensuring at the beginning of the experiment the absolute majority in cell numbers on the side of the prey organism. This was done to avoid coincidences due to simple nutrient competition.

### 2.5. Experimental Design

#### 2.5.1. Isolation

For the isolation of bacteriolytic strains, dilutions of samples (undiluted, 1:10, 1:100) from the lake were poured on the bacterivory test media, which contained a living cell lawn of *A. citreus*. This was followed by the incubation of the plates for 5 days at 30 °C in the dark to inhibit growth of phototrophic microorganisms. Promising strains showed their ability to feed on the prey bacteria by producing clear lysis zones (Figure S1 in Supplementary Materials). An inoculation loop was then used to transfer the strain from the middle of the lysis zone to nutrient agar.

Different dilutions of the samples from the lake were used to avoid the possibility of the too rapid overgrowth of the plates in the case of high organism numbers, no clearly visible lysis zones and thus then no isolation of pure cultures of bacteriolytic strains.

#### 2.5.2. Identification Methods

Cell morphology was examined using a phase contrast microscope (Axiolab, Zeiss, Singapore). The Gram characteristics were determined using a 3% (*w*/*v*) KOH-solution [28]. The presence of catalase was detected with a 3% (*v*/*v*) hydrogen peroxide solution, and Bactident-Oxidase stripes (Merck) were used to verify the presence of cytochrome-c-oxidase. For the Paenibacillus apiarius, type strain physiological properties were investigated according to Gordon et al. (1973) [29].

Matrix assisted laser desorption ionization–time of flight mass spectrometry was conducted using a Microflex L20 mass spectrometer (Bruker Daltonics, Billerica, MA, UnUSA) equipped with a N_2_ laser as described by Tóth et al. (2008) [30]. The spectra of strains SBUG 1926, SBUG 1927, SBUG 1941 and SBUG 1947 were identified by the MALDI Biotyper software (Version 3.1) using the 2014 database update V4.0.0.1.

Automated Ribotyping was performed by the RiboPrinter System (DuPont Qualicon, Wilmington, DE, USA) using the restriction enzyme *Eco*RI according to published protocols [31].

Genomic DNA extraction for strain SBUG 1947 was performed with the MasterPure™ Gram Positive DNA Purification Kit from Epicentre (Madison, WI, USA). Amplification of the 16S rRNA gene with primer 27f and 1525r was carried out as described by Rainey et al. (1996) [32]. Purified PCR products were partially sequenced using the BigDye^®^ Terminator v1.1 Cycle Sequencing Kit and the Applied Biosystems 3500xl Genetic Analyzer (Waltham, MA, USA). The resulting sequences were analyzed using the EzTaxon webtool [33,34]. SBUG 1947 has been deposited as DSM 28162 in the German Collection of Microorganisms and Cell Cultures (Leibniz-Institute DSMZ).

#### 2.5.3. Screening of the Isolates for Bacteriolytic and Bacterivorous Capability on Solid Lysis Media

To confirm bacterivorous activity and to determine the prey range, the bacteriolytic isolates were tested on solid bacterivory media with the living preys *A. citreus*, *M. luteus*, *Pseudomonas putida* and *E. coli* each in three parallel experiments. The testing of bacteriolytic capability against autoclaved cells was carried out using these same prey bacteria autoclaved before use at 121 °C for 21 min. A consistent small quantity of isolated cell material, grown for 24 h on nutrient agar, was applied to the lawn of living or autoclaved prey bacteria with an inoculation loop. Incubation was for 5 days at 30 °C. To quantify lytic activities, the diameters of the clearing zones on the lawns were measured with a caliper.

#### 2.5.4. Analysis of Bacterivorous Capability in Co-Culture

*P. veronii* SBUG 1927 and *P. apiarius* SBUG 1947 were examined for their ability to feed on living cells of *A. citreus* in a nutrient-poor liquid medium. In the co-cultures, 25 mL of the prey bacterium cell suspension of *A. citreus* (OD_500_ = 0.2) was mixed with 25 mL of the cell suspension of either *P. veronii* SBUG 1927 or *P. apiarius* SBUG 1947 (OD_500_ = 0.05). As control cultures, the cell suspensions of the prey and each of the bacterivorous isolates were supplemented with 25 mL of DNB alone. The co-cultures and the controls were incubated in 500 mL Erlenmeyer flasks at 30 °C for 24 h. To evaluate the changes in the cell concentrations over 24 h, growth curves were created by direct colony counting on nutrient agar plates.

#### 2.5.5. Analysis of Bacterivorous Capability in Co-Culture of *P. apiarius* SBUG 1947 for Enrichment of the Novel Antimicrobial Substance

The simulated deficiency situation was intended to stimulate the isolate *P. apiarius* SBUG 1947 to secrete antimicrobial and/or lytic substances against the test strain *A. citreus* SBUG 321 so that these could later be analytically detected in the culture supernatant. First, both the test strain and the isolate were pre-cultured on nutrient agar plates for 48 h and then transferred to nutrient broth II using an inoculation loop. They were incubated for 24 h to achieve a sufficiently high cell density for co-culture. Subsequently, the cell suspensions were centrifuged (Sorvall^®^ RC-5B Refrigerated Superspeed Centrifuge, Du Pont Instruments 5000× *g* rpm 4 °C 8 min) to separate the cells from the culture medium and transfer the cell pellets into the DNB. The determination of the optical density (spectrophotometer Thermo Scientific, Genesys 20 at a wavelength of 500 nm in cuvettes with a layer thickness of 1 cm) was used to adjust the inoculation amounts. For this purpose, the suspension of the isolate *P. apiarius* SBUG 1947 was adjusted to an OD_500_ of 0.05 and that of the test strain *A. citreus* SBUG 321 to an OD_500_ of 0.2. In order to obtain as much culture supernatant as possible after cultivation, these experiments were performed in a 10 L Erlenmeyer flask. In total, 500 mL of each of the suspensions was added to the 10 L Erlenmeyer flask, and the co-culture was incubated for 48 h. At 12 h intervals, 250 mL of each suspension was removed, centrifuged (Sorvall^®^ RC-5B Refrigerated Superspeed Centrifuge, Du Pont Instruments 8000× *g* rpm 4 °C 8 min) and then sterile filtered (Sarstedt Filtropur V 50 vacuum filter, 0.22 µm pore size) to ensure that no cells remained in the culture supernatant. The culture supernatant of this co-culture is abbreviated from now on as PaAcCoCu (*P. apiarius A. citreus* Co-Culture). In addition, suspensions of *A. citreus* and *P. apiarius* (cell density as described above) were prepared. To each of them was added 250 mL of DNB, and these control cultures were incubated in 5 L Erlenmeyer flasks for 48 h. Again, samples (250 mL of the suspension) were taken at 24 h intervals, centrifuged and sterile filtered as described above. These control cultures are abbreviated below as AcC (*A. citreus* Control), PaC (*P. apiarius* Control) and for the control of the culture medium DNBC (Diluted Nutrient Broth Control).

### 2.6. Lyophilization of Culture Supernatant

The centrifuged and sterile filtered culture supernatants were lyophilized prior to storage. For this purpose, the liquid samples were first frozen for 24 h at −20 °C and then overnight at −70 °C in crystallization dishes. Subsequently, the samples were dried under a pressure of 1.013 mbar and 20 °C in the lyophilization unit (Alpha 1–4; Christ, Osterode, Germany). The substances of the culture supernatant (proteins, peptones, carbohydrates and substances secreted by the bacteria) remain in the crystallizing dishes as a dried, durable and stable powder. In the following, this is referred to as freeze-dried culture supernatant.

### 2.7. Solid Phase Extraction of Culture Supernatant

Immediately prior to analytical measurements and prior to fractionations by preparative high-performance liquid chromatography, the culture supernatants were subjected to pre-purification by solid phase extraction (SPE). For this purpose, a vacuum pump (Vacuubrand^®^ MZ 2 C, Wertheim, Germany) was used in conjunction with a vacuum box (J.T. Baker spe-12G, Krün, Germany) and LiChrolut RP C18 columns (Merck^®^, Darmstadt, Germany). Non-polar and moderately polar substances are retarded on the column due to interactions with the stationary phase, whereas carbohydrates and salts remaining in the aqueous phase pass through the column without interaction and are discarded.

The column was first equilibrated using 5 mL of dH_2_O and 5 mL of methanol. Then, the culture supernatant dissolved in dH_2_O was applied. The flow through was discarded, and retained substances were recovered using 5 mL methanol.

### 2.8. Isolation of a Bacteriolytic Substance via Preparative High-Performance Liquid Chromatography

For the analyses to determine the exact active substance of the culture supernatant, the individual components of the culture supernatant had to be separated beforehand. Since the supernatant is a highly complex mixture of substances, preparative high-performance liquid chromatography was applied. The Agilent Technologies 1260 Infinity system from Agilent (Santa Clara, CA, USA) was used for this purpose: System 1260 Infinity consisting of G1364B—1260 FC-PS fraction collector, G1315D—1260 DAD VL diode array detector, G2260A—1260 Prep ALS autosampler, G1361A—1260 prep pump, column: Eclipse XDB-C18, 21.2 × 250 mm; 7 µm, (Agilent). The fractionation of 200 µL culture supernatant into eight fractions was achieved by a flow rate of 10 mL/min, a gradient of the following: 0 min = 25% MeOH, 5 min to 75% MeOH, 8.5 min to 85% MeOH and 9 min to 100% MeOH and with the following fractions result: first fraction (#1): 4.2–5.8 min, second fraction (#2): 5.9–7.2 min, third fraction (#3): 7.3–8.0 min, fourth fraction (#4): 8.1–9.6 min, fifth fraction (#5): 9.9–11.1 min, sixth fraction (#6): 11.7–12.3 min, seventh fraction (#7): 12.4–12.9 min, eighth fraction (#8): 13.0–16.8 min.

We performed seven fractionations on one day with 100 mg culture supernatant solved in 1.5 mL dH_2_O. Hence, each fractionation contained approximately 14.3 mg of the culture supernatant PaC for 48 h. The solution was purified by SPE (cf. Section 2.7) and then separated using the fractionation scheme as described above. The respective fractions were collected in Erlenmeyer flasks and later concentrated to dryness in a rotary vacuum evaporator (VACUU-BRAND^®^ PC 3001 VARIO™) for long-term storage in the fridge.

### 2.9. Analytical Methods for the Detection and Structural Characterization of Active Substances

#### 2.9.1. Coupling of Liquid Chromatography with Tandem Mass Spectrometry

For the chemical detection of antimicrobial active substances, liquid chromatography coupled with tandem mass spectrometry was initially used. A liquid chromatography system from Thermo Scientific (Bremen, Germany) with the following configuration was used for the analyses: Thermo Scientific Easy nLC 1000—AB Sciex Triple ToF 5600 Tandem mass spectrometer, column: self-made with C18 material from an “Aeris-Peptide” column, injection volume: 1 µL, flow rate: 20 µL/min, gradient: 0–3 min from 1% to 5% acetonitrile with 0.1% acetic acid, 3–69 min linear up to 25% acetonitrile with 0.1% acetic acid, 70–79 min to 75% acetonitrile with 0.1% acetic acid, up to 80 min to 99% acetonitrile with 0.1% acetic acid.

For this measurement, 70 µL methanol was added to each 5 mg of the freeze-dried culture supernatant, and the dissolved upper phase (after 30 min) was removed, transferred to a sample vial and evaporated overnight. Finally, the dried culture supernatant was taken up in 70 µL distilled water.

#### 2.9.2. Coupling of Liquid Chromatography, Mass Spectrometry and Ultraviolet–Visible Spectroscopy

For the separation of the individual substances by preparative high-performance liquid chromatography (cf. Section 2.8), the absorption spectra in the ultraviolet–visible range were also required. This was made possible by the diode array detector of the liquid chromatography coupled with mass spectrometry and ultraviolet–visible spectroscopy system. For the detection of the individual substances separated by liquid chromatography coupled with mass spectrometry and ultraviolet–visible spectroscopy, the same solvent gradient described in Section 2.8 (liquid chromatography coupled with tandem mass spectrometry measurement) was used. Only the chromatography column differed from the one described in Section 2.8. An Agilent Technologies (Santa Clara, CA, USA) 1200 series instrument with attached 6120 quadrupole mass spectrometer was used for the analyses: pre-column: Zobrax XDB C8, column: Zobrax XDB C18, 4.6 × 150 mm, 5µm, Agilent, flow rate: 400 µL/min, injection volume: 4 µL, gradient: see in Section 2.8.

These liquid chromatography coupled with mass spectrometry and ultraviolet–visible spectroscopy analyses were performed using freeze-dried culture supernatants (2 mg) on the one hand, and the fractions were separated by preparative high-performance liquid chromatography on the other hand.

#### 2.9.3. Structure Elucidation by Nuclear Magnetic Resonance Spectroscopy

For the structural elucidation of the active compound, one and two-dimensional nuclear magnetic resonance spectra (^1^H NMR—Proton (H) Nuclear Magnetic Resonance, HSQC—Heteronuclear Single-Quantum Correlation, HMBC—Heteronuclear Multiple Bond Correlation, ^13^C NMR—Carbon-13 (C13) Nuclear Magnetic Resonance, and ^1^H^1^H COSY—Proton (H) Proton (H) homonuclear correlation spectra) were recorded. Fraction #5 was recorded on a Bruker Avance 600 instrument (Rheinstetten, Germany) at 600 MHz. The solvents of the different measurements were DMSO-*d*_6_ and D_2_O. Chemical shifts are expressed in δ (ppm) calibrated on the resonances of the residual non-deuterated solvent. *J* values are given in Hz.

### 2.10. Testing the Antimicrobial Activity of Freeze-Dried Culture Supernatants on Double-Layer Agar Plates

To test the respective culture supernatants (PaC, AcC, PaAcCoCu at different time points and the DNBC) for antimicrobial activity, 2 mg were weighed into a micro reaction vessel (Eppendorf AG, Hamburg, Germany) and dissolved in either 50 µL methanol or 20 µL dH_2_O. The use of both solvents should provide initial information about the solubility of the culture supernatants and possible active substances contained therein.

In order to be able to make statements about the presence of antimicrobial active substances, Rotilabo^®^ antibiotic paper discs were used as standard. These were first autoclaved at 121 °C for 20 min and transferred to sterilized glass Petri dishes for the respective tests. Subsequently, the corresponding dissolved culture supernatants were pipetted onto them. To avoid solvent-induced bactericidal effects on the double-layer agar plates, especially by methanol, the antibiotic paper discs were dried under a sterile hood for at least 20 min before being transferred individually to the cell-containing double-layer agar plates. The appropriate drying time, as described above, was determined when establishing the test method using antibiotic paper discs wetted with pure dH_2_O or methanol. As a negative control, these were applied to the cell-containing double-layer agar plates after the 20 min drying period and also incubated as described above. To ensure better diffusion, all plates were first stored in the refrigerator at 5 °C for 4 h before incubation, and then incubated at 30 °C for 48 h. Finally, the inhibition zone radii were determined for the plates using a caliper. Therefore, the measurement started from the edge of the antibiotic disc (the diameter of the antibiotic disc itself was excluded) to the periphery of the inhibition zone (Figure S2 in Supplementary Materials).

### 2.11. Testing the Antimicrobial Activity of Fractions #1 to #8 and of the Isolated Antimicrobial Substance on Double-Layer Agar Plates

To test the fractions separated by preparative high-performance liquid chromatography for their antimicrobial efficacy, the double-layer agar plates (cf. Section 2.3 and Section 2.10) containing the test organism *Arthrobacter citreus* were used. The eight fractions, dried-down by a rotary evaporator, stored at 4 °C, solved in dH_2_O or MeOH, were finally pipetted in two different concentrations onto the Rotilabo^®^ antibiotic sterile paper discs (cf. Section 2.10). Concentration C1 corresponded to 2 mg culture supernatant per paper disc and concentration C2 corresponded to double this amount—i.e., approximately 4 mg culture supernatant per antibiotic paper disc and double-layer agar plate. Subsequently, the antibiotic paper discs were dried to avoid effects of the solvent, placed on the double-layer agar plates, initially stored at 4 °C for 4 h in the refrigerator and measured after 48 h of incubation at 30 °C using a caliper.

### 2.12. Electron Microscopy

For visualization by electron microscopy, cells of *A. citreus* SBUG 321 and *P. apiarius* SBUG 1947 as well as a co-culture with both organisms were cultivated in DNB, and samples were taken at various times (after 1 h and 16 h) during cultivation.

For scanning electron microscopy, the cells were separated from the culture medium by filtration through a 0.2 μm pore size polycarbonate filter. Cells adsorbed to the filter were fixed with a solution of 1% glutaraldehyde, 4% paraformaldehyde and 50 mM NaN_3_ in 5 mM HEPES (pH 7.4) and stored at 4 °C until further processing. Samples were then washed with washing buffer (100 mM cacodylate buffer, 1 mM CaCl_2_; pH 7) three times for 5 min each, treated with 2% tannic acid in washing buffer for 1 h, 1% osmium tetroxide in washing buffer for 1 h, 1% thiocarbohydrazide for 30 min, 1% osmium tetroxide in washing buffer at 4 °C overnight, and 2% uranyl acetate in 0.9% sodium chloride solution for 30 min—with washing steps in between. After dehydration in a graded series of aqueous ethanol solutions (10, 30, 50, 70% for 15 min each, and 80, 90, 96% for 10 min each) and a final treatment with 100% ethanol three times for 10 min each, samples were critical point-dried with liquid CO_2_. Finally, filters with the adsorbed cells were mounted on aluminum stubs, sputtered with gold/palladium and examined with a scanning electron microscope EVO LS10 (Carl Zeiss Microscopy GmbH, Oberkochen, Germany). Afterwards, all micrographs were edited by using Adobe Photoshop.

## 3. Results

### 3.1. Isolation and Identification of Bacteriolytic Strains

Four bacterial strains that are bacteriolytic against living *A. citreus* on solid lysis test media were isolated from the brackish water site Strait Uzynaral of Lake Balkhash (Table 2).

Strains SBUG 1926, SBUG 1927 and SBUG 1941 were identified by matrix assisted laser desorption ionization–time of flight mass spectrum analyses as members of the *Pseudomonas fluorescens* group and showed highest score values (2.429, 2.478 and 2.371, respectively) for the database entries *Pseudomonas veronii* B 561 UFL (SBUG 1926 and 1927) and *Pseudomonas veronii* B 559 UFL (SBUG 1941) (MSP dendrogram Figure S3 in Supplementary Materials). Score values >2.3 indicate a “highly probable species identification” according to the Biotyper 3.1 User Manual (Bruker Daltonics). The *Eco*RI RiboPrint patterns of strains SBUG 1926 and SBUG 1927 belonged to the same RiboGroup. Strains belonging to the same RiboGroup cannot be differentiated by the RiboPrinter System and should be regarded as identical. Although there was no automated identification, the patterns of strains SBUG 1926 and SBUG 1927 showed the highest similarity (0.83) to the entry of *Pseudomonas veronii* B 561 UFL. Strain SBUG 1947 was tentatively identified as *Paenibacillus apiarius* by matrix assisted laser desorption ionization–time of flight mass spectrometry with a score of 1.834 to the database entry for *Paenibacillus apiarius* DSM 5581^T^ (“probable genus identification”). Though strain SBUG 1947 was not identified automatically by the RiboPrinter System, the entry of the DuPont Identification Library for *Paenibacillus apiarius* DSM 5581^T^ showed the highest similarity (0.77) to its *Eco*RI band pattern. A partial sequencing of the 16S-rDNA gene resulted in an unambiguous assignment to the species *Paenibacillus apiarius* (Figure S4 in Supplementary Materials). The hylogenetic dendrogram indicated the position of strain SBUG 1947 within the genus *Paenibacillus*. The evolutionary history was inferred by using the Maximum Likelihood method based on the General Time Reversible (GTR) model. A discrete Gamma distribution was used to model evolutionary rate differences among sites. Evolutionary analyses were conducted in MEGA7 [35]. The sequence of strain SBUG 1947 was identical to tate of the type strain *Paenibacillus apiarius* DSM 5581, and strain SBUG 1947 was deposited at DSMZ under the number DSM 28162. The type strain *Paenibacillus apiarius* DSM 5581^T^ and our isolate strain SBUG 1947 are both positive for nitrate reduction, urea degradation, casein hydrolysis and acid formation from glucose. The four identified bacteriolytic strains are catalase-positive, rod-shaped and are Gram-negative in the quick test. *Paenibacillus apiarius* SBUG 1947 is oxidase-negative, while strains *Pseudomonas veronii* SBUG 1926, SBUG 1927 and SBUG 1941 are oxidase-positive. All strains were isolated from water samples with alkaline pH values of about 8, so they are at least alkali-tolerant.

### 3.2. Bacteriolysis Activity and Bacterivory of Isolated Strain

All three isolated strains of *Pseudomonas veronii*, as well as *Paenibacillus apiaries*, formed clearing zones on cell lawns of living *A. citreus* cells with moderate lysis activity and a constant zone size (Table 2). Lysis zone ranges on solid media with living and autoclaved cells of *Pseudomonas putida*, *E. coli*, *M. luteus* and *A. citreus* were measured after five days of incubation at 30 °C (Table 3; examples for plate images Figures S5–S9 in Supplementary Materials).

All isolates showed lysis activity against all living prey species. Only living cells of *Pseudomonas putida* were not lysed by *Pseudomonas veronii* SBUG 1927 and SBUG 1941. The isolates were, in general, not able to lyse the autoclaved cells of the Gram-positive Actinobacteria except for *P. apiarius* SBUG 1947, which degraded autoclaved cells of *M. luteus*. The strains SBUG 1927, SBUG 1926 and SBUG 1947 had strong lysis activity against the autoclaved Gram-negative Proteobacteria used, while strain SBUG 1941 did not lyse any of the autoclaved prey cell species.

### 3.3. Bacteriolysis against Arthrobacter Citreus in Co-Culture

**Figure 2:**
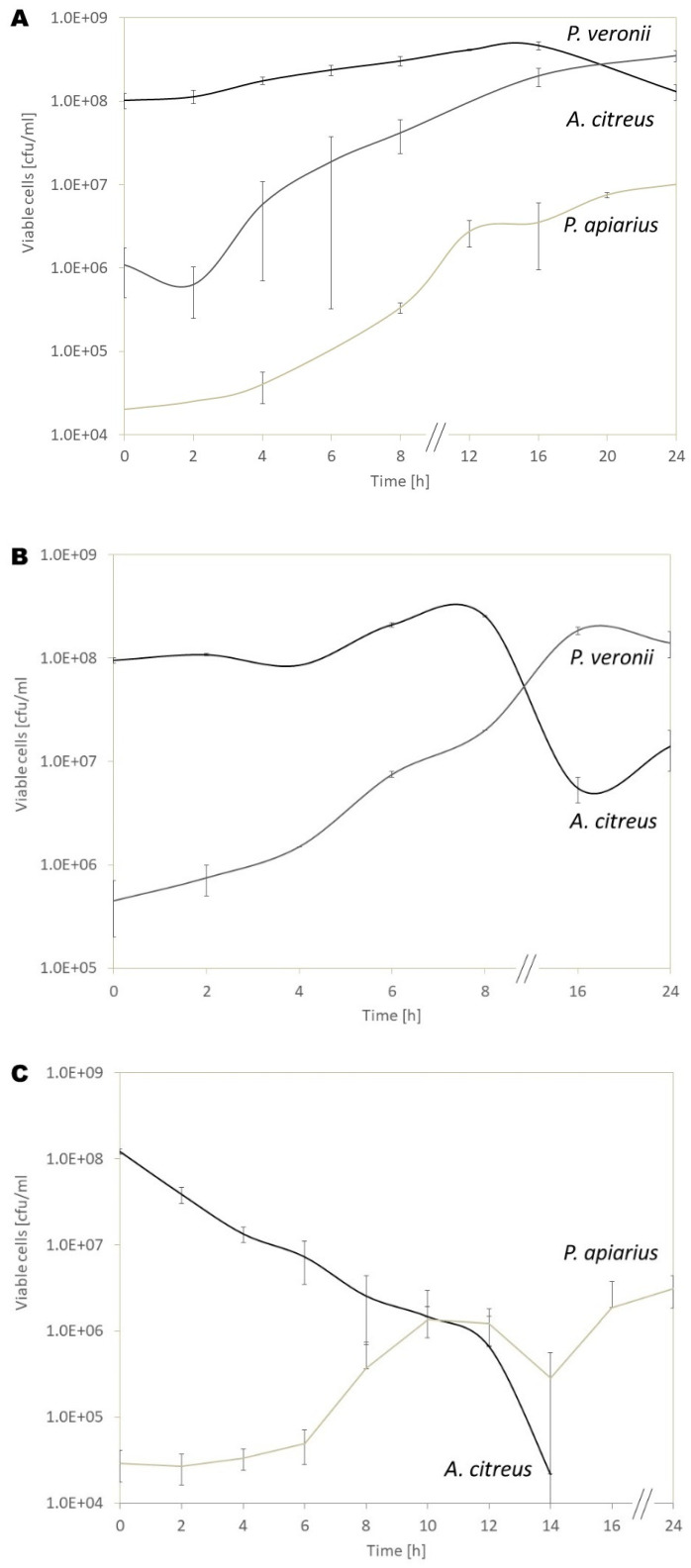
Growth curves of pure and co-cultures in DNB (tenfold diluted nutrient broth) for 24 h at 30 °C and 130 rpm. The initial OD_500_ of *A. citreus* was set to 0.2, while it was set to 0.05 for *P. veronii* and *P. apiarius* (**A**) Growth of the three separate control cultures of *A. citreus* SBUG 321, *P. veronii* SBUG 1927 and *P. apiarius* SBUG 1947 (**B**) Growth of *P. veronii* SBUG 1927 and *A. citreus* SBUG 321 in a co-culture (**C**) Growth of *P. apiarius* SBUG 1927 and *A. citreus* SBUG 321 in a co-culture.

In order to characterize the bacteriolysis process in the DNB, growth curves were recorded for the co-cultures of *A. citreus* supplemented with the bacteriolytic isolates *P. veronii* SBUG 1927 or *P. apiarius* SBUG 1947 (Figure 2A–C).

**Figure 3:**
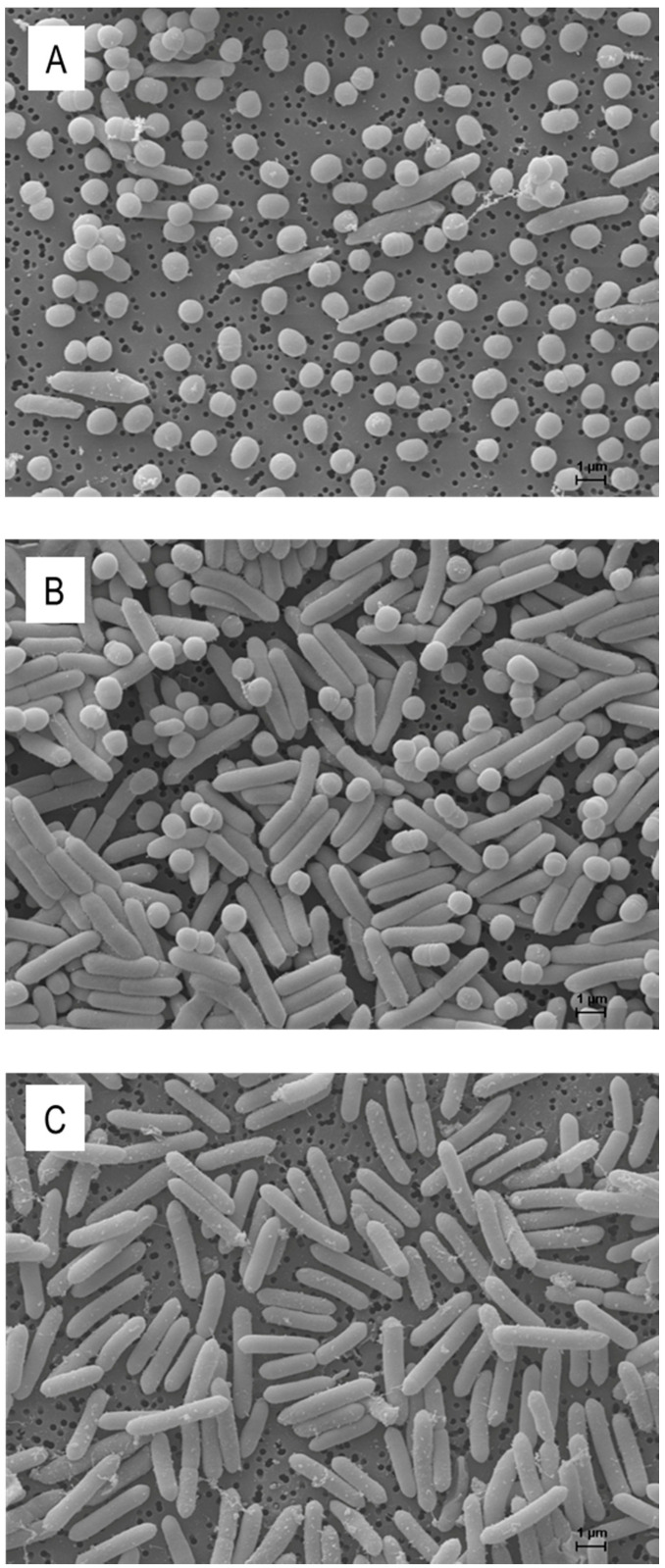
Scanning electron micrographs of *Arthrobacter citreus* and *Paenibacillus apiarius* grown in co-culture within DNB for 1 h (**A**) and for 16 h (**B**) as well as the pure culture of *Paenibacillus apiarius* after 16 h of cultivation (**C**). Scale bar = 1 µm.

During the co-cultivation of *P. apiarius* SBUG 1947 and *A. citreus*, samples were taken at different time points to visualize the bacteriolytic activity within the liquid culture by scanning electron microscopy (Figure 3). The micrographs demonstrate that the ratio of cells to each other reversed within 16 h. While *A. citreus* cells predominated at the beginning (Figure 3A), *P. apiarius* cells dominated after 16 h (Figure 3B). Even though the CFU could no longer be determined at this time point (Figure 2C), *A. citreus* cells were still present (Figure 3B). However, there were hardy any lysed cells visible.

The initial OD_500_ of *A. citreus* in the co-culture was set to 0.1 to reach a cell number of 9 × 10^7^ to 1 × 10^8^ colony-forming units (cfu) mL^−1^. The initial OD_500_ of the bacteriolytic isolates was set to 0.025, resulting in somewhat different cfu numbers of the individual strains (see below).

#### 3.3.1. Co-Culture of *P. veronii* SBUG 1927 and *A. citreus*

The initial number of viable cells of *P. veronii* was 9 × 10^5^ cfu mL^−1^ (Figure 2A,B). In the control culture without prey cells, the viable cell number of *P. veronii* increased to 3 × 10^8^ cfu mL^−1^ after 24 h of incubation in DNB (Figure 2A). The number of colony-forming units of *A. citreus* inoculated with higher initial OD_500_ reached its maximum with 4 × 10^8^ cfu mL^−1^ after 16 h and decreased thereafter to a certain extent (Figure 2A). In the nutrient-poor DNB medium *A. citreus* does not grow exponentially, and the cell number stays more or less constant in the pure culture.

In co-culture, the *P. veronii* cells first grew, corresponding to the control cultures for 8 h of incubation (Figure 2B). During the first 8 h of co-cultivation, the viable cell number of *A. citreus* also increased with an extended generation time of 6 h 15 min, which is around 2 h longer than its generation time in the control culture (Table 4).

Between 8 and 16 h of co-culture, the number of viable *A. citreus* cells decreased abruptly while *P. veronii* was in its exponential growth phase. The minimum of viable *A. citreus* was reached after 17 h and was followed by a slowly increasing cfu number. After 24 h of incubating the co-culture, the viable cell number of *A. citreus* was still reduced to one-tenth compared to the pure culture control.

#### 3.3.2. Co-Culture of *P. apiarius* SBUG 1947 and *A. citreus*

At the beginning of the culture experiments, the number of viable cells of *P. apiarius* was 2 × 10^4^ cfu mL^−1^ using an initial OD_500_ of 0.025 (Figure 2A). The maximum number of cfu in the *P. apiarius* control culture was reached after 24 h with 1 × 10^7^ cfu mL^−1^. Compared to the control, *P. apiarius* did not grow within the first 6 h of incubation in the co-culture, and the cell number after 24 h was only 4 × 10^6^ cfu mL^−1^ (Figure 2C). In the co-culture, the number of living *A. citreus* decreased from the beginning and reached a minimum of 6.5 × 10^4^ cfu mL^−1^ after 14 h of co-cultivation in DNB. After 14 h, the few colonies of *A. citreus* were overgrown by *P. apiarius* on the nutrient agar plates, rendering agar plate counting impossible from that time on. The generation time of *A. citreus, at* −1 h 27 min, was the same negative value as for the co-culture with *P. veronii* (Table 4). The electron micrographs validate the decrease of coccoid *A. citreus* cells in comparison to *P. apiarius* (Figure 3).

### 3.4. Antimicrobial Activity of Different Culture Supernatants of P. apiarius SBUG 1947

Since the lysis of *A. citreus* was clearly demonstrated for strain *P. apiarius* SBUG 1947 by co-culture and microscopic methods, the culture supernatant of *P. apiarius* SBUG 1947 in co-culture and in pure culture was examined for its antimicrobial activity.

#### 3.4.1. Agar Diffusion Tests of Different Culture Supernatants of *P. apiarius* SBUG 1947 on Double Layer Agar Plates with *A. citreus*

The culture supernatant of the *P. apiarius* control formed inhibition zones on double layer agar plates with *A. citreus*. In addition, the inhibition zone radii of the culture supernatant dissolved in dH_2_O (A) were on average about 30% larger than those dissolved in MeOH (B) (Table 5; plate images Figures S10–S15 in Supplementary Materials).

Neither the culture supernatant of *A. citreus* (AcC) nor the culture medium (DNBC) had inhibitory effects, and thus these served as negative controls.

The culture supernatant of the co-culture—*P. apiarius* and *A. citreus* (PaAcCoCu)—as well as that of the *P. apiarius* control (PaC) contained substances that formed inhibition zones on plates with *A. citreus*. Furthermore, the inhibition zones increased with increasing cultivation time of the co-culture, from which we concluded an increasing concentration of the active substance(s). Only in the 12 h co-cultures were no inhibition zones visible, while the size of the inhibition zones increased with increasing cultivation time of the co-culture. This suggests an increasing accumulation of the active substances during culture. The inhibition zone radii of the culture supernatant dissolved in dH_2_O (A) were always larger than those dissolved in methanol (B). It is also striking that after 24 h of cultivation, the inhibition zones of the co-culture were much smaller than those of the *P. apiarius* control after 24 h without any contact to the prey organism.

#### 3.4.2. Agar Diffusion Tests of Culture Supernatants of *P. apiarius* SBUG 1947 on Double Layer Agar Plates with Different Test Strains

Since the inhibition zones of the co-culture were much smaller, only the culture supernatant of the *P. apiarius* control after 48 h (PaC 48 h) was used for agar diffusion tests with different test strains. The culture medium (DNBC) was included as a negative control. Initially, strains of the risk group 1 (low individual and low community risk; these microorganisms are unlikely to cause disease) were tested: *A. citreus*, *Micrococcus luteus*, *Pseudomonas putida*, *Escherichia coli* and *Bacillus subtilis* (Table 6).

An inhibition “yard-forming” effect of the culture supernatant of the *P. apiarius* is only evident with the Gram-positive test strains *A. citreus*, *M. luteus* and *B. subtilis*. However, it is striking that the inhibition zone radii formed by the culture supernatant of *P. apiarius* after 48 h cultivation (PaC 48 h) on double layer agar plates with *A. citreus* are on average 28% smaller than those formed with the culture supernatant after 24 h cultivation (PaC 24 h) (Table 5 and Table 6). The growth of the Gram-negative test strains *Pseudomonas putida* and *E. coli* was not inhibited in any of the assays.

In addition to the test strains of risk group 1, the following strains of risk group 2 (moderate individual risk, limited community risk; these microorganisms are unlikely to be a significant risk to laboratory workers or the environment, but exposure may cause infection) were also tested: *Staphylococcus aureus*, *Pseudomonas aeruginosa*, *Listeria monocytogenes*, *Enterococcus faecalis* and *Bacillus cereus* (Table 6). In these assays, the efficacy of the culture supernatant *P. apiarius* was also limited exclusively to the Gram-positive test strains *S. aureus* and *B. cereus*, with the effect on *S. aureus* with inhibition zones around 8 mm being much stronger than on *B. cereus* with 2.6 mm. In the case of *S. aureus*, the inhibition zones were even larger than those of *A. citreus.* Unfortunately, the test strains *L. monocytogenes* and *E. faecalis* could not be assayed since they failed to grow on the bactopepton agar.

### 3.5. Detection, Isolation, Characterization and Structure Elucidation of an Antimicrobially Active Substance in Culture Supernatants of P. apiarius SBUG 1947

After the agar diffusion tests (Table 5 and Table 6) had demonstrated antimicrobial effects of the culture supernatants of *P. apiarius* SBUG 1947, the analytical detection of possible antimicrobial substances was carried out with liquid chromatography coupled with tandem mass spectrometry. First, culture supernatants of *P. apiarius*, the *A. citreus* control and the culture medium control were examined to detect substances that were exclusively found in the culture supernatant of *P. apiarius*. In the superimposed elution profiles of the culture supernatant of *P. apiarius* and the controls of *A. citreus* and the culture medium, a peak was detected at 75.2 min, which was exclusively found in the culture supernatant of *P. apiarius* (Figures S16 and S17 in Supplementary Materials). This peak at 75.2 min was termed PP1. In addition to the qualitative detection of PP1 in the culture supernatant of *P. apiarius* by liquid chromatography, the molecular weight of PP1 was determined by tandem mass spectrometry as 422 Da ([M + H]^+^ 423.2473) (Figure S18 in Supplementary Materials).

**Figure 4:**
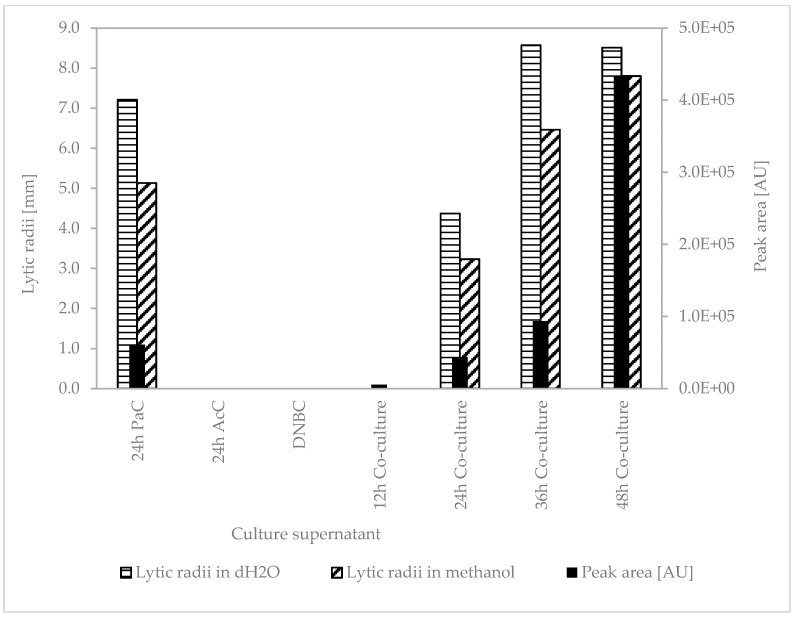
Peak areas measured by liquid chromatography coupled with tandem mass spectrometry of PP1 in culture supernatants compared to antimicrobial (lytic) activity of PP1 in different culture supernatants of *P. apiarius* SBUG 1947 against *A. citreus*.

We then looked for the PP1 signal in the elution profiles of the culture supernatants of the co-cultures of *P. apiarius* and *A. citreus* after 12 h, 24 h, 36 h and 48 h and checked whether the peak areas of PP1 correlated with the increasing inhibition zone radii described in Table 5 (Figure 4).

Peak PP1 at 75.2 min is clearly detected in all elution profiles of *P. apiarius* SBUG 1947 supernatants, and the mass spectra of these peaks also correspond to that of PP1. In addition, there is a clear increase in the intensity of peak PP1 with the increasing cultivation time of the co-cultures. This suggests that the concentration of the compound increases with increasing cultivation time. At the 12 h time point, there are about 5.5 × 10^3^ peak area units, which are not yet sufficient for visible antimicrobial activity, while after 48 h of cultivation, there are 4.3 × 10^5^ peak area units, and strong antimicrobial activity was detected. In the control of *P. apiarius*, the peak area is also quite high, 6.08 × 10^4^, which is approximately between the co-cultures after 24 and 36 h. In the two negative controls—AcC 24 h and DNBC—peak PP1 was not detected, and in parallel, no antimicrobial activity was observed.

To isolate PP1 by preparative high-performance liquid chromatography, we required its ultraviolet–visible spectrum. This was determined using a liquid chromatography coupled with mass spectrometry and ultraviolet–visible spectroscopy system with an adapted method (Figure S19 in Supplementary Materials). PP1′s molecular weight of 422.2 Da was confirmed, and the ultraviolet–visible maxima were determined as 210 nm, 245 nm and 315 nm by liquid chromatography coupled with mass spectrometry and ultraviolet–visible spectroscopy (Figure S20 in Supplementary Materials).

To separate PP1 from the other substances, the culture supernatant was separated into eight fractions using preparative high-performance liquid chromatography (cf. Section 2.8), with fraction #5 containing the PP1 as the main component (Figures S21 and S22 in Supplementary Materials). Firstly, 100 mg of the freeze dried culture supernatant (PaC 48 h) was used for the solid phase extraction to get rid of carbohydrates and salt. The liquid output (solved in MeOH) was completely dried and then solved (unweighted) in 1.5 mL dH_2_O. This volume was sufficient for seven exact fractionations (one measuring day) with preparative high-performance liquid chromatography; thus, each of the seven fractionation contained 200 µL of the solid phase extraction solution. The output volume of every fraction depends on the period of time in the fractionation scheme (Figure S6 in Supplementary Materials). For the fraction of interest #5, which was collected from 9.9 min up to 11.1 min (exact 72 s with flow rate of 10 mL/min), we collected 12 mL of the fraction. Due to the fact that these 12 mL contained approximately 66 µg (calculated) of solid matter, we did not weigh every single fractionation, but we weighed the output of 42 fractionations. This corresponds to 6 measuring days, and here, we obtained an output of 2.76 mg dry matter of fraction #5 containing the PP1 as main component (Figure S7 in Supplementary Materials).

For the further structural elucidation of PP1, NMR measurements were performed—^1^H NMR, HSQC, HMBC, ^13^C NMR and ^1^H^1^H COSY in different solvents DMSO-*d*_6_ and D_2_O. All data led to the compilation of NMR results in Table 7.

**Figure 5:**
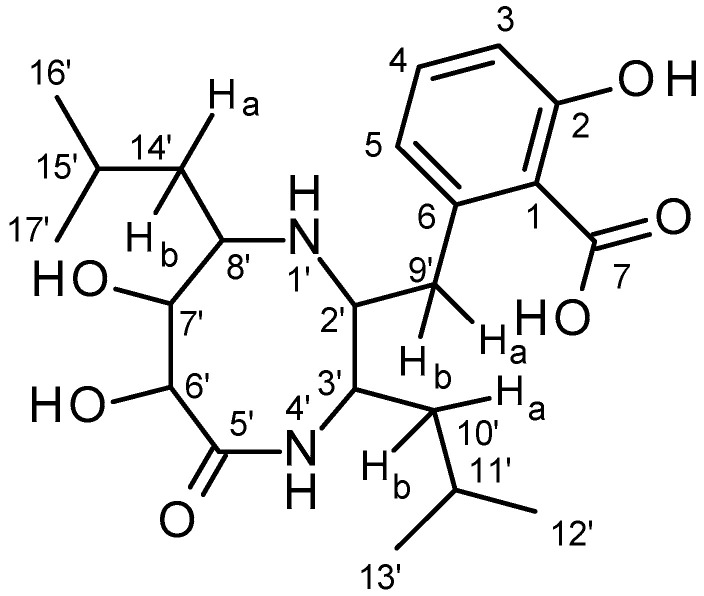
Structure hypothesis of the antimicrobially active substance PP1: 6-[(6,7-dihydroxy-3,8-diisobutyl-5-oxo-1,4-diazocan-2-yl)methyl]-2-hydroxy-benzoic acid.

The evaluation of all nuclear magnetic resonance and mass spectrum data led to the structure hypothesis of 6-[(6,7-dihydroxy-3,8-diisobutyl-5-oxo-1,4-diazocan-2-yl)methyl]-2-hydroxy-benzoic acid for PP1 (Figure 5).

### 3.6. Agar Diffusion Tests of Fractionated Culture Supernatants of P. apiarius SBUG 1947

To determine whether the antimicrobial activity of the culture supernatants of *P. apiarius* SBUG 1947 was indeed exclusively due to product PP1, the preparative high-performance liquid chromatography fractions #1 to #8 were tested on double layer agar plates as described in Section 2.11.

For this purpose, fractions #1 to #8 were again dissolved in methanol, pipetted on the antibiotic discs, dried for at least 20 min and placed in the middle of the double layer agar plates. The test was performed in two different concentrations. Concentration C1 corresponded to the amount of PP1 used in the previous agar diffusion tests with the lyophilized culture supernatants (2 mg), and concentration C2 represented a doubling of C1 (Table 8).

These results finally clarified that PP1 in fraction #5 is the substance with the inhibition yard-forming effect and that the antimicrobial activity is also exclusively attributable to this substance. In addition, it was to be determined whether the substance PP1 contained in fraction #5 can also induce inhibition zones on the other tested bacterial strains *M. luteus* and *B. subtilis* as well as *S. aureus* and *B. cereus*. For this purpose, fraction #5 dissolved in methanol was pipetted onto the antibiotic paper discs as described in Section 2.11 and then evaporated, and these were placed on simple inoculation strips of the additional test strains. After 48 h of cultivation, growth inhibitions of the test bacteria were recognizable for all four tested strains around the antibiotic paper discs. Further away from the antibiotic paper discs, the cells of test bacteria showed normal growth. This also confirmed the antimicrobial effect of PP1 on the other Gram-positive strains.

## 4. Discussion

### 4.1. Bacteriolysis on Solid Agar Media

The isolates *P. veronii* SBUG 1927 and *P. apiarius* SBUG 1947 from brackish water of Lake Balkhash in Kazakhstan showed clear lysis zones of moderate sizes against autoclaved and living prey cells. However, compared with *B. pumilis* [3] isolated under similar conditions, the measured lysis zones of *P. veronii* SBUG 1927 and *P. apiarius* SBUG 1947 against autoclaved cells were on average only half as large. In this *B. pumilis* strain, mainly antibacterial metabolites such as diketopiperazines and pumilacidins were described, to which the antibacterial activity was assigned [36,37]. Since neither diketopiperazines nor pumilacidins were found for *P. veronii* SBUG 1927 and *P. apiarius* SBUG 1947, and comparatively good lytic activities were also detected against living cells, it was initially hypothesized that the clear zones with small diameters might probably indicate the secretion of lytically active enzymes, which are less diffusible than bacteriolytic antibiotics because of their higher molecular weight, we therefore suggest that *P. veronii* and *P. apiarius* secrete rather bacteriolytic enzymes than antibiotic substances. The lysis test results on autoclaved prey cells partly align with this proposal. Against autoclaved cells of *M. luteus* and *A. citreus*, there was no bacteriolytic activity detected for all strains of *P. veronii* although the living cells of both prey species were lysed (Table 3). In conclusion, the initial step of cell wall degradation, which is the prerequisite for the following lysis process, is the specific attack and destruction of the living cell by the bacterivorous strains.

### 4.2. Bacteriolysis in Liquid Co-Culture with A. citreus

#### 4.2.1. Bacteriolysis of *A. citreus* by *P. veronii* SBUG 1927

The strain SBUG 1927 was identified as *P. veronii*. The first strains of this species isolated from mineral water were decribed by Elomari et al. in 1996 [38]. To our knowledge, predatory effects have not been previously described for *P. veronii* though they have been for other species of the genus *Pseudomonas*. For example, the nonobligate predatory *Pseudomonas* strain 679-2 attaches to prey cells and produces a toxic growth initiation factor and an unidentified antibacterial and antifungal compound [39]. Furthermore, many *Pseudomonas* spp. produce surfactant lipopeptides such as viscosins, amphisins, tolaasins and syringomycins [40]. *Pseudomonas* spp. are also known to produce murein hydrolases [41]. *Pseudomonas aeruginosa* releases bacteriolytic membrane vesicles filled with such peptidoglycan hydrolases and the antibiotic gentamicin. The vesicles can fuse with the outer membrane of Gram-negative bacteria or adhere to the cell wall of Gram-positive bacteria leading to their lysis [42]. *Pseudomonas aeruginosa* additionally possesses the bacteriolytic muramidase effector Tse3, which can be injected into the periplasm of neighboring bacterial preys or competitor cells by a Type VI secretion apparatus [43].

In our study, the bacteriolysis of *A. citreus* commenced after 8 h of co-cultivation when *P. veronii* SBUG 1927 enters the stationary growth phase, suggesting that the bacteriolytic behavior of this species is probably quorum dependent. After 16 h, the number of cfu of *A. citreus* does not further decrease, suggesting that the bacteriolysis of *P. veronii* is a temporary phenomenon rather than durable predation.

#### 4.2.2. Bacteriolysis of *A. citreus* by *P. apiarius* SBUG 1947

The species *Paenibacillus apiarius* was first isolated from honeybee larvae in 1955 by Katznelson and described as “*Bacillus apiarius*” [44]. Later, it was reclassified to the genus *Paenibacillus* [45]. Little is known about the bacteriolytic capabilities of *P. apiarius*. The present study shows strong bacteriolytic activity of *P. apiarius* against *A. citreus* on solid agar media as well as in liquid media. In a liquid co-culture with both bacterial species in DNB, the cell number declined steadily (Figure 2C, Table 4). This decline is commensurate with the electron microscopic data (Figure 3), although there was no evidence for cell–cell-contact between *P. apiarius* and its prey. Thus, cell–cell contact might not be necessary, and instead soluble lysis factors may play a role during the bacteriolysis process. Hints of such antagonistic activities are known for other species of the genus *Paenibacillus*. For instance, *P. alvei* produces two membrane active polypeptide antibiotics, named paenibacillin P, with activity against Gram-positive bacteria and paenibacillin N with activity against Gram-negative bacteria [46]. Moreover, as the *Pseudomonas* group, *Paenibacillus tianmuensis* is able to produce several bacteriolytic lipopeptides—for instance, battacin [47].

### 4.3. Antimicrobial Activity of Culture Supernatants of P. apiarius SBUG 1947

The aim of the series of tests of culture supernatants of *P. apiarius* SBUG 1947 was to clarify whether the bacteriolytic and/or antimicrobial capabilities of the isolate *P. apiarius* in liquid culture with *A. citreus* requires direct cell–cell contact or whether it involves the secretion of active substances.

The agar diffusion tests of culture supernatants of the co-culture—*P. apiarius* and *A. citreus* (PaAcCoCu)—as well as of that of the *P. apiarius* control (PaC) on double layer agar plates with *A. citreus* (Table 5) showed clearly that the inhibition zones increased with increasing cultivation time of the co-culture and that the *P. apiarius* control also showed corresponding inhibition zones, suggesting that direct cell–cell contact of the two strains is not essential and rather suggesting the secretion of antimicrobial active substances. The test results of the *P. apiarius* control after 48 h (PaC 48 h) with the other test strains *M. luteus*, *Pseudomonas putida*, *E. coli* and *B. subtilis* (Table 6) also fit this notion. The formation of extracellular antibacterial metabolites has also been described for other bacteria of the genus *Paenibacillus*. Thus, the lantibiotics paenibacillin produced by *Paenibacillus polymyxa* [48,49] and paenibacillin N and P produced by *Paenibacillus alvei* [46], the polypeptide antibiotic AN 5-1 also produced by *Paenibacillus alvei* [50] and the pelgipeptines A & B produced by *Paenibacillus elgii* [51] showed antibacterial activities against various species. In addition, our strain *P. apiarius* SBUG 1947 and its culture supernatants showed antibacterial activities against several Gram-positive species.

### 4.4. Characterization of the Antimicrobial Active Substance PP1 of Culture Supernatants of P. apiarius SBUG 1947

Fractionation by preparative high-performance liquid chromatography yielded eight fractions, of which only fraction #5 contained the metabolite PP1, and only this fraction was effective against *A. citreus* in antimicrobial tests (Table 8). A part of the lyophilizate of fraction 5 was dissolved in DMSO-*d*6 and another part in D_2_O, and both samples were analyzed by NMR (Table 7). We proposed the hypothetical structure of 6-[(6,7-dihydroxy-3,8-diisobutyl-5-oxo-1,4-diazocan-2-yl)methyl]-2-hydroxy-benzoic acid for PP1 (Figure 5). This structure consists on the one hand of an aromatic ring and on the other hand of a saturated nitrogen and oxygen-containing heterocyclic system. With the carboxyl group at the C-1 atom and the hydroxyl group at the C-2 atom, the aromatic ring resembles so-called salicylic acid, now known under the trade name Aspirin. This drug was introduced in 1898 by the Bayer company and is the world’s best-selling anti-inflammatory painkiller [52]. Salicylic acid also has antimicrobial activity against *Propionibacterium acnes* and is sometimes used in cosmetic products [53]. In the work of Gershon and Parmegiani (1962) [54], various derivatives of salicylic acid tested positive against *Aerobacter aerogenes*, *Leuconostoc mesenteroides*, *Streptococcus faecalis* (now *Enterococcus faecalis*) and *Staphylococcus aureus*. Furthermore, anacardic acid, also a derivative of salicylic acid, also has antimicrobial activity, even against methicillin-resistant *Staphylococcus aureus* [55].

The saturated nitrogen and oxygen-containing heterocyclic system resembles, on the one hand, the diketopiperazines described for other *Bacillus* strains. On the other hand, it also resembles lipopeptides such as pumilacidins, surfactins and bacircines, especially because of the two branched aliphatic substituents—the two isobutyl groups attached at the C-3′ and the C-8′ atoms (Figure 5). Diketopiperazines are cyclic peptides derived from two α-amino acids forming six-membered rings containing two cis-peptide bonds [56], and antimicrobial effects have been ascribed to them [36,57,58,59]. In addition to Gram-positive test bacteria, also Gram-negative bacteria such as *Loktanella hongkongensis*, *Ruegeria* sp. [58], *Escherichia coli* [60] and also fungi such as *Candida albicans* and *Pyricularia oryzae* [57,61] were growth-inhibited by diketopiperazines.

The lipopeptides pumilacidins, surfactins and bacircines are amphiphilic biotensides that can reduce surface tension and penetrate lipid bilayers. In bacteria such as *Pseudomonas* sp., *Bacillus* sp., and *Streptomyces* sp. and also in fungi, they are widespread and are involved in biocontrol. Pumilacidins from *B. pumilus* [62,63,64,65], surfactins from *Bacillus amyloliquefaciens* [66,67], from *Bacillus subtilis* [68,69] and from *Bacillus* spp. [70,71] as well as bacircines from *B. pumilus* isolated from a marine sponge *Ircina* sp. [63,72] are well described. The lipopeptide extracts of *Bacillus pumilus* containing pumilacidin A to G lysed living cells of *A. citreus* and *M. luteus*, suggesting that lipopeptides can be the key initial factor for the bacteriolytic process [37]. In addition to these lipopeptides, the so-called AMPs (antimicrobial peptides) have also been extensively studied [73], especially with regard to their antibacterial effects [74,75,76].

## 5. Conclusions

Predators or prokaryotes able to produce bacteriolytic substances in general can be an important resource for natural product research. In the present study, we isolated four bacterial strains from brackish water samples collected from Lake Balkhash in Kazakhstan, an extremely remote area in Central Asia. The sequencing of the 16S rRNA gene identified the strain SBUG 1947 as *Paenibacillus apiarius*, while the strains SBUG 1926, SBUG 1927 and SBUG 1941 were identified by matrix assisted laser desorption ionization–time of flight mass spectrometry as members of the *Pseudomonas fluorescens* group, with high similarity to *Pseudomonas veronii*.

The examination of bacteriolytic capabilities of these strains revealed striking effects in particular on living prey cells on solid media, as well as in liquid co-culture with *A. citreus*. Growth curves and electron micrographs documenting the bacteriolysis process in co-cultures of *P. apiarius* SBUG 1947 and its prey *A. citreus* SBUG 321 gave us an idea of the time course of the predatory behavior. We observed a disappearance of prey cells in this co-culture within 16 h and found a temporal correlation to the production of a novel antibiotic substance. We propose this antibiotic substance PP1 to be 6-[(6,7-dihydroxy-3,8-diisobutyl-5-oxo-1,4-diazocan-2-yl)methyl]-2-hydroxy-benzoic acid with structural similarities to the anti-inflammatory painkiller salicylic acid. As we see once again, microorganisms from natural habitats are a constant resource for new substances with useful potential in pharmacy and biotechnology.

The effectiveness and physicochemical stability of the substance PP1 paved the way for further investigation potential and a broad variety of applications in green chemistry and drug research.

## Figures and Tables

**Table 1:** Sampling sites and sampling conditions of water samples A–E from Strait Uzynaral, Lake Balkhash (Kazakhstan).

Sample	A	B	C	D	E
Region	Lake Balkhash, Strait Uzynaral, North Coast				
Location	100 m in front of the reed belt	100 m from A (east)	100 m from A (west)	100 m in front of A (south)	100 m in front of B (south)
Sampling date	11 November 2012	11 November 2012	11 November 2012	11 November 2012	11 November 2012
Temperature water surface (°C)	6	6	6	6	6
Salinity (%)	0.153	0.154	0.145	0.171	0.156
pH	8.7	8.5	8.8	8.9	8.8
Sample amount (mL)	10	10	10	10	10

**Table 2:** Lysis activity on living cells of *Arthrobacter citreus* SBUG 321 on solid agar media. Colony morphology of the bacteriolytic isolates from different sampling sites of Lake Balkhash after 24 h of incubation; the strains were later identified as *Paenibacillus apiarius* (SBUG 1947) and *Pseudomonas veronii* (SBUG 1926, SBUG 1927 and SBUG 1947).

Sampling Site	Strain	Lysis Zones (mm) on *A. citreus*	Colonial Characteristics			
			Form	Color	Surface	Elevation
B	SBUG 1926	1.2	±circular	cream	smooth	raised
C	SBUG 1927	1.0	±circular	cream	smooth	raised
D	SBUG 1941	0.7	circular	cream	smooth	raised
D	SBUG 1947	1.3	irregular	white	smooth	raised

Sampling sites according to Table 1.

**Table 3:** Bacteriolysis activity of *Pseudomonas veronii* SBUG 1926, SBUG 1927, SBUG 1941 and *Paenibacillus apiarius* SBUG 1947 against autoclaved (A) and living (L) cells of the Gram-negative strains *Pseudomonas putida* and *Escherichia coli* as well as Gram-positive *Micrococcus luteus* and *Arthrobacter citreus* after five days of incubation at 30 °C.

	Bacteriolysis Activity							
	*P. veronii* SBUG 1926		*P. veronii* SBUG 1927		*P. veronii* SBUG 1941		*P. apiarius* SBUG 1947	
	A	L	A	L	A	L	A	L
Proteobacteria								
*Pseudomonas putida*	+++	++	+++	+	−	−	+++ ^(a)^	++
*Escherichia coli*	+++	++	+++	++	−	+	+++	++ ^(b)^
Actinobacteria								
*Micrococcus luteus*	−	++	−	++	−	+	++	++
*Arthrobacter citreus*	−	++ ^(c)^	−	++ ^(d)^	−	++	−	++ ^(e)^

Lysis activity against (A) autoclaved/(L) living indicator cells; − (no lysis activity): 0–0.2 mm diameter of the lysis zone; + (low): 0.2–0.5 mm diameter of the lysis zone; ++ (moderate): 0.5–1.5 mm diameter of the lysis zone; +++ (strong): >1.5 mm diameter of the lysis zone; (a) plate image Figure S5 in Supplementary Materials, (b) plate image Figure S6 in Supplementary Materials, (c) plate image Figure S7 in Supplementary Materials, (d) plate image Figure S8 in Supplementary Materials (e) plate image Figure S9 in Supplementary Materials.

**Table 4:** Generation times of *A. citreus* in the control culture, during growth and lysis phase in the co-culture with *P. veronii* and in co-culture with *P. apiarius*.

Parameter	Control	Co-Culture with *P. veronii*		Co-Culture with *P. apiarius*
		Growth Phase (0–8 h)	Lysis Phase (8–16 h)	
Initial cell number (cfu mL^−1^)	8.50 × 10^7^	1.00 × 10^8^	2.55 × 10^8^	1.20 × 10^8^
Achieved cell number (cfu mL^−1^)	3.25 × 10^8^	2.55 × 10^8^	5.50 × 10^6^	2.53 × 10^6^
Generation time t_gen_ (h:min)	4:10	6:15	−1:27	−1:27

**Table 5:** Antimicrobial activity of different culture supernatants of *P. apiarius* SBUG 1947 against *A. citreus*.

Type of Culture Supernatant	Number of Image in Supplementary Materials	Inhibition Zone Radius (mm)	
		A (dH_2_O)	B (MeOH)
PaC (24 h)	-	7.2 ± 1.1	5.1 ± 0.4
AcC (24 h) (control)	Figure S10	0 ± 0	0 ± 0
DNBC (control)	Figure S11	0 ± 0	0 ± 0
PaAcCoCu (12 h)	Figure S12	0 ± 0	0 ± 0
PaAcCoCu (24 h)	Figure S13	4.4 ± 0.8	3.2 ± 0.6
PaAcCoCu (36 h)	Figure S14	8.6 ± 0.7	6.5 ± 0.6
PaAcCoCu (48 h)	Figure S15	8.5 ± 0.5	7.8 ± 0.4

**Table 6:** Antimicrobial activity of culture supernatants of *P. apiarius* SBUG 1947 against different test strains of risk groups 1 and 2.

**Type of Culture Supernatant**	**Inhibition Zone Radius (mm)**				
	**Strains of the Risk Group 1**				
	*A. citreus*	*M. luteus*	*B. subtilis*	*E. coli*	*Pseudomonas putida*
PaC (48 h)	5.2 (±1)	10.5 (±1)	6.3 (±0.7)	0 (±0)	0 (±0)
DNBC (control)	0 (±0)	0 (±0)	0 (±0)	0 (±0)	0 (±0)
	**Strains of the risk Group 2**				
	*S. aureus*	*B. cereus*	*L. mono-cytogenes*	*E. faecalis*	*Pseudomonas aeruginosa*
PaC (48 h)	8.0 (±0.5)	2.6 (±0.5)	ng	ng	0 (±0)
DNBC (control)	0 (±0)	0 (±0)	ng	ng	0 (±0)

ng = no growth of the test strains.

**Table 7:** ^1^H assignments, HSQC, HMBC and ^1^H^1^H COSY correlations for PP1. (^1^H NMR—Proton (H) Nuclear Magnetic Resonance, HSQC—Heteronuclear Single-Quantum Correlation, HMBC—Heteronuclear Multiple Bond Correlation, ^13^C NMR—Carbon-13 (C13) Nuclear Magnetic Resonance, and ^1^H^1^H COSY—Proton (H) Proton (H) homonuclear correlation).

^1^H Assignments	HSQC Correlations	HMBC Correlations	^1^H^1^H COSY Correlations
7.92 (s(broad), NH, H-1′) ^(a)^	-	-	-
7.75 (d, J = 9.4 Hz, 1H, NH, H-4′)	-	(39.3 (C-10′)) ^(b)^, 47.9 (C-3′), 173.2 (C-5′)	4.20 (H-3′)
7.47 (dd, J = 7.8 Hz, 1H, H-4)	136.2 (C-4)	(108.3 (C-1)), (115.3 (C-3)), 140.7 (C-6), 160.8 (C-2)	6.82 (H-5), 6.85 (H-3)
6.85 (d, J = 8.1 Hz, 1H, H-3)	115.3 (C-3)	108.3 (C-1), 118.4 (C-5), (140.7 (C-6)), (160.8 (C-2)), (169.0 (C-7))	6.82 (H-5), 7.47 (H-4)
6.81 (d, J = 7.0 Hz, 1H, H-5)	118.4 (C-5)	29.1 (C-9′), 108.3 (C-1), 115.3 (C-3), (136.2 (C-4)), (140.7 (C-6)),	(3.04 (H_a_-9′)), 6.85 (H-3), 7.48 (H-4)
4.69 (d, J = 12.8 Hz, 1H, H-2′)	81.1 (C-2′)	(29.1 (C-9′)), (39.3 (C-10′)), 140.7 (C-6)	(2.86 (H_b_-9′)), 3.04 (H_a_-9′), (4.20 (H-3′))
4.20 (t, J = 9.4 Hz, 1H, H-3′)	47.9 (C-3′)	(23.6 (C-11′)), (29.1 (C-9′)), 39.3 (C-10′), (173.2 (C-5′))	(1.33 (H-10′)), 1.67 (H-11′), (4.69 (H-2′)), 7.75 (NH, H-4′)
4.01 (d, J = 4.9 Hz, 1H, H-6′)	73.4 (C-6′)	51.4 (C-8′), 74.6 (C-7′), 173.2 (C-5′)	3.46 (H-7′)
3.46 (s(broad), 1H, H-7′)	74.6 (C-7′)	(173.2 (C-5′))	2.82 (H-8′), 4.01 (H- H-6′)
3.04 (d, J = 16.0 Hz, 1H, H_a_-9′)	29.1 (C-9′)	(47.9 (C-3′)), 81.1 (C-2′), (108.3 (C-1)), (118.4 (C-5)), 140.7 (C6),	(4.69 (H-2′)), 2.86 (H_b_-9′)
2.86 (d, J = 16.0 Hz, 1H, H_b_-9′)	29.1 (C-9′)	(47.9 (C-3′)), 108.3 (C-1), 118.4 (C-5), 140.7 (C-6),	(4.69 (H-2′)), 3.03 (H_a_-9′)
2.82 (m, 1H, H-8′)	51.4 (C-8′)	-	3.46 (H-7′)
1.70 (m, J = 10.9 Hz, 1H, H_a_-10′)	39.3 (C-10′)	21.3 (C-12′/C-13′), 47.9 (C-3′)	1.33 (H_b_-10′)
1.74 (m, 1H, H-15′)	23.4 (C-15′)	-	0.86 (H-16′/H-17′)
1.67 (d, J = 8.8 Hz, 1H, H-11′)	23.6 (C-11′)	21.3 (C-12′/C-13′), (39.3 (C-10′)), 47.9 (C-3′), (81.1 (C-2′))	4.20 (H-3′),1.33 (H_b_-10′), 0.89 (H-12′/H-13′)
1.37 (m, J = 10.9 Hz, 1H, H_a_-14′)	40.9 (C-14′)	23.4 (C-15′)	1.17 (H_b_-14′)
1.33 (m, J = 10.9 Hz, 1H, H_b_-10′)	39.3 (C-10′)	(21.3 (C-12′/C-13′),), 23.6 (C-11′), (47.9 (C3′))	4.20 (H-3′), 1.70 (H_a_-10′)0.89 (H-12′/H-13′)
1.17 (m, J = 10.9 Hz, 1H, H_b_-14′)	40.9 (C-14′)	21.5 (C-16′/C-17′), (23.4 (C-15′)), 51.4 (C-8′)	1.37 (H_a_-14′), 1.74 (H-15′), 2.82 (H-8′),
0.89 (d, J = 6.2 Hz, H-12′/H-13′)	24.3 (C-12′/C-13′)	21.3 (C-12′/C-13′)	1.33 (H-11′)
0.86 (m(t), J = 7.2 Hz, J = 6.5 Hz, 6H, H-16′/H-17′)	21.5 (C-16′/C-17′)	21.5 (C-16′/C-17′), 23.4 (C-15′), 40.9 (C-14′), (51.4 (C-8′))	1.74 (H-15′)
0.77 (d, J = 6.2 Hz, H-12′/H-13′)	21.3 (C-12′/C-13′)	23.6 (C-10′), 24.3 (C-12′/C-13′), 39.3 (C-11′)	1.33 (H-11′)

^(a)^ Chemical shifts are expressed in *d* (ppm) calibrated on the resonances of the residual nondeuterated solvent DMSO. *J* values are in Hz. ^(b)^ Signals with low intensity.

**Table 8:** Antimicrobial activity of fraction #1 to #8 of culture supernatants of *P. apiarius* SBUG 1947 against *A. citreus*.

Number of Fractions of Culture Supernatant	Inhibition Zone Radius (mm)	
	Concentration C1	Concentration C2
#1	0 (±0)	0 (±0)
#2	0 (±0)	0 (±0)
#3	0 (±0)	0 (±0)
#4	0 (±0)	0 (±0)
#5	4.5 (±0.2)	6.2 (±0.9)
#6	0 (±0)	0 (±0)
#7	0 (±0)	0 (±0)
#8	0 (±0)	0 (±0)

## Supplementary Materials

The following supporting information can be downloaded at: https://www.mdpi.com/article/10.3390/microorganisms10081519/s1, Figure S1: Enlarged photography of a clear lysis zone on a lawn of pregrown and living *A. citreus* cells, Figure S2: Inhibition radii and caliper, Figure S3: MSP dendrogram generated on the basis of the matrix assisted laser desorption ionization–time of flight mass spectra from proteins (BioTyper software, Bruker) showing the diversity of *Pseudomons veronii* strains and the isolated strains SBUG 1926, SBUG 1927 and SBUG 1941, Figure S4: Phylogenetic dendrogram indicating the position of strain SBUG 1947 within the genus *Paenibacillus*. Bootstrap values ≥50% are indicated at branch points. Bar, 0.05 substitutions per nucleotide position, Figure S5: Image lytic radii *P. apiarius* SBUG 1947 on autoclaved cells of *P. putida*, Figure S6: Image inhibition radii *P. apiarius* SBUG 1947 on living cells of *E. coli*, Figure S7: Image inhibition radii *P. veronii* SBUG 1926 on living cells of *A. citreus*, Figure S8: Image inhibition radii *P. veronii* SBUG 1927 on living cells of *A. citreus*, Figure S9: Image inhibition radii *P. apiarius* SBUG 1947 on living cells of *A. citreus*, Figure S10: Image of the freeze-dried culture supernatant of *A. citreus* 24 h (negative control) on living cells of *A. citreus*, Figure S11: Image of the freeze-dried culture supernatant of diluted nutrient broth (negative control) on living cells of *A. citreus*, Figure S12: Image of the freeze-dried culture supernatant of *P. apiarius* and *A. citreus* co-culture 12 h (PaAcCoCu (12 h)) on living cells of *A. citreus*, Figure S13: Image of the freeze-dried culture supernatant of *P. apiarius* and *A. citreus* co-culture 24 h (PaAcCoCu (24 h)) on living cells of *A. citreus*, Figure S14: Image of the freeze-dried culture supernatant of *P. apiarius* and *A. citreus* co-culture 36 h (PaAcCoCu (36 h)) on living cells of *A. citreus*, Figure S15: Image of the freeze-dried culture supernatant of *P. apiarius* and *A. citreus* co-culture 48 h (PaAcCoCu (48 h)) on living cells of *A. citreus*, Figure S16: Liquid chromatography coupled with tandem mass spectrometry elution profiles of the culture supernatants of *A. citreus* (blue line), *P. apiarius* (red line) and the control of the culture medium (pink line), Figure S17: Detailed section of the liquid chromatography coupled with tandem mass spectrometry elution profile from Figure S16 between 74 and 78 min, *A. citreus* (blue line), *P. apiarius* (red line) and the control of the culture medium (pink line), Figure S18: Mass spectrum [M + H]^+^ of PP1 measured by tandem mass spectrometry, Figure S19: Liquid chromatography coupled with mass spectrometry elution profile of the co-culture supernatants of *P. apiarius* and *A. citreus*, Figure S20: Mass spectrum [M + H]^+^ and ultraviolet–visible spectrum of PP1 measured by liquid chromatography coupled with mass spectrometry and ultraviolet–visible spectroscopy, Figure S21: Fractionation scheme of preparative high-performance liquid chromatography with fractions #1–#8 (from left to right), Figure S22: Liquid chromatography coupled with mass spectrometry and ultraviolet–visible spectroscopy elution profile of fraction #5 containing PP1 as main compound and mass spectrum of PP1 confirming the molecular weight of 422.2 Da.
