# Supplementary material for: A Novel Antimicrobial Metabolite Produced by Paenibacillus apiarius Isolated from Brackish Water of Lake Balkhash in Kazakhstan
Source: Microorganisms. 2022 Jul 27;10(8):1519. doi: 10.3390/microorganisms10081519 (PMC9416454; doi:10.3390/microorganisms10081519)
Supplement: Supplementary file 1 [file microorganisms-10-01519-s001.zip › microorganisms-1751239-supplementary.pdf]

Supplementary Materials

A novel antimicrobial metabolite produced by *Paenibacillus apiarius* isolated from brackish water of Lake Balkhash in Kazakhstan

Alexander Meene[1,#], Christiane Herzer [1,#], Rabea Schlüter[2], Bolatkhan Zayadan[3], Ruediger Pukall[4], Peter Schumann[4], Frieder Schauer[1], Tim Urich[1], Annett Mikolasch[1,*]

[1] University Greifswald, Institute of Microbiology, Felix-Hausdorff-Straße 8, 17487 Greifswald, Germany
e-mail: annett.mikolasch@uni-greifswald.de
[2] University Greifswald, Imaging-Center of the Department of Biology, Friedrich-Ludwig-Jahn-Str. 15,
17487 Greifswald, Germany
[3] Al-Farabi Kazakh National University, Department of Biotechnology, 71, al-Farabi ave, 050040 Almaty, Kazakhstan
[4] Leibniz Institute DSMZ - German Collection of Microorganisms and Cell Cultures, Inhoffenstrasse 7B
38124 Braunschweig, Germany
# These authors contributed equally to the work (Shared first author)
* Correspondence: annett.mikolasch@uni-greifswald.de; Tel.: +49 (0)3834-4205917

Figure S1: Enlarged photography of a clear lysis zone on a lawn of pregrown and living *A. citreus* cells

Figure S2: Inhibition radii and caliper

Figure S3: MSP dendrogram generated on the basis of the matrix assisted laser desorption ionization-time of flight mass spectra from proteins (BioTyper software, Bruker) showing the diversity of *Pseudomons veronii* strains and the isolated strains SBUG 1926, SBUG 1927 and SBUG 1941

Figure S4: Phylogenetic dendrogram indicating the position of strain SBUG 1947 within the genus *Paenibacillus*. Bootstrap values ≥50% are indicated at branch points. Bar, 0.05 substitutions per nucleotide position

Figure S5: Image lytic radii *P. apiarius* SBUG 1947 on autoclaved cells of *P. putida*

Figure S6: Image inhibition radii *P. apiarius* SBUG 1947 on living cells of *E. coli*

Figure S7: Image inhibition radii *P. veronii* SBUG 1926 on living cells of *A. citreus*

Figure S8: Image inhibition radii *P. veronii* SBUG 1927 on living cells of *A. citreus*

Figure S9: Image inhibition radii *P. apiarius* SBUG 1947 on living cells of *A. citreus*

Figure S10: Image of the freeze-dried culture supernatant of *A. citreus* 24 h (negative control) on living cells of *A. citreus*

Figure S11: Image of the freeze-dried culture supernatant of diluted nutrient broth (negative control) on living cells of *A. citreus*

Figure S12: Image of the freeze-dried culture supernatant of *P. apiarius* and *A. citreus* co-culture 12 h (PaAcCoCu (12 h)) on living cells of *A. citreus*

Figure S13: Image of the freeze-dried culture supernatant of *P. apiarius* and *A. citreus* co-culture 24 h (PaAcCoCu (24 h)) on living cells of *A. citreus*

Figure S14: Image of the freeze-dried culture supernatant of *P. apiarius* and *A. citreus* co-culture 36 h (PaAcCoCu (36 h)) on living cells of *A. citreus*

Figure S15: Image of the freeze-dried culture supernatant of *P. apiarius* and *A. citreus* co-culture 48 h (PaAcCoCu (48 h)) on living cells of *A. citreus*

Figure S16: Liquid chromatography coupled with tandem mass spectrometry elution profiles of the culture supernatants of *A. citreus* (blue line), *P. apiarius* (red line) and the control of the culture medium (pink line)

Figure S17: Detailed section of the liquid chromatography coupled with tandem mass spectrometry elution profile from Figure S16 between 74 and 78 min, *A. citreus* (blue line), *P. apiarius* (red line) and the control of the culture medium (pink line)

Figure S18: Mass spectrum $[M+H]^+$ of PP1 measured by tandem mass spectrometry

Figure S19: Liquid chromatography coupled with mass spectrometry elution profile of the co-culture supernatants of *P. apiarius* and *A. citreus*

Figure S20: Mass spectrum $[M+H]^+$ and ultraviolet–visible spectrum of PP1 measured by liquid chromatography coupled with mass spectrometry and ultraviolet–visible spectroscopy

Figure S21: Fractionation scheme of preparative high-performance liquid chromatography with fractions #1 - #8 (from left to right)

Figure S22: Liquid chromatography coupled with mass spectrometry and ultraviolet–visible spectroscopy elution profile of fraction #5 containing PP1 as main compound and mass spectrum of PP1 confirming the molecular weight of 422.2 Da

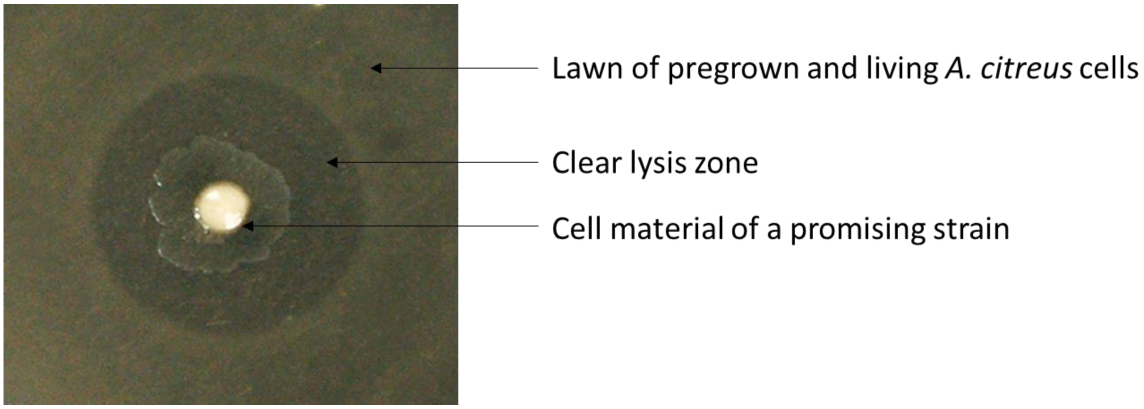

Figure S1 Enlarged photography of a clear lysis zone on a lawn of pregrown and living *A. citreus* cells.

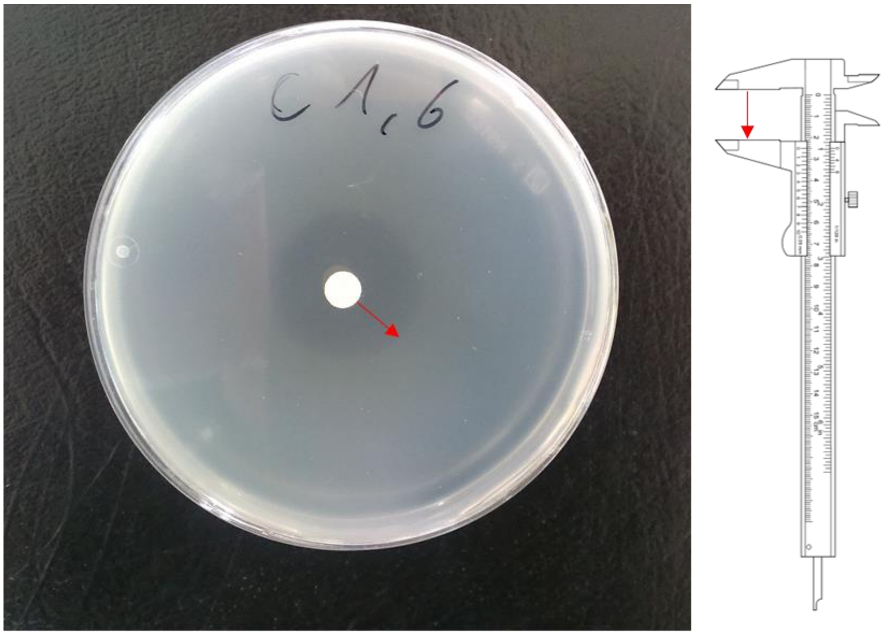

Figure S2 Inhibition radii and caliper.

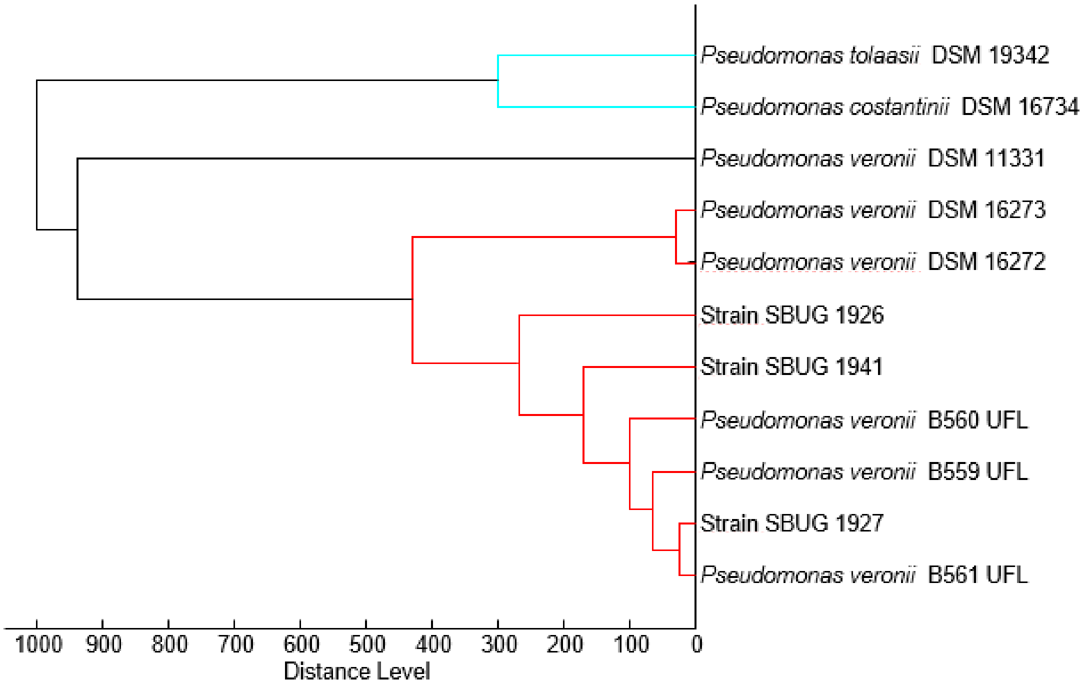

Figure S3 MSP dendrogram generated on the basis of the matrix assisted laser desorption ionization-time of flight mass spectra from proteins (BioTyper software, Bruker) showing the diversity of *Pseudomons veronii* strains and the isolated strains SBUG 1926, SBUG 1927 and SBUG 1941.

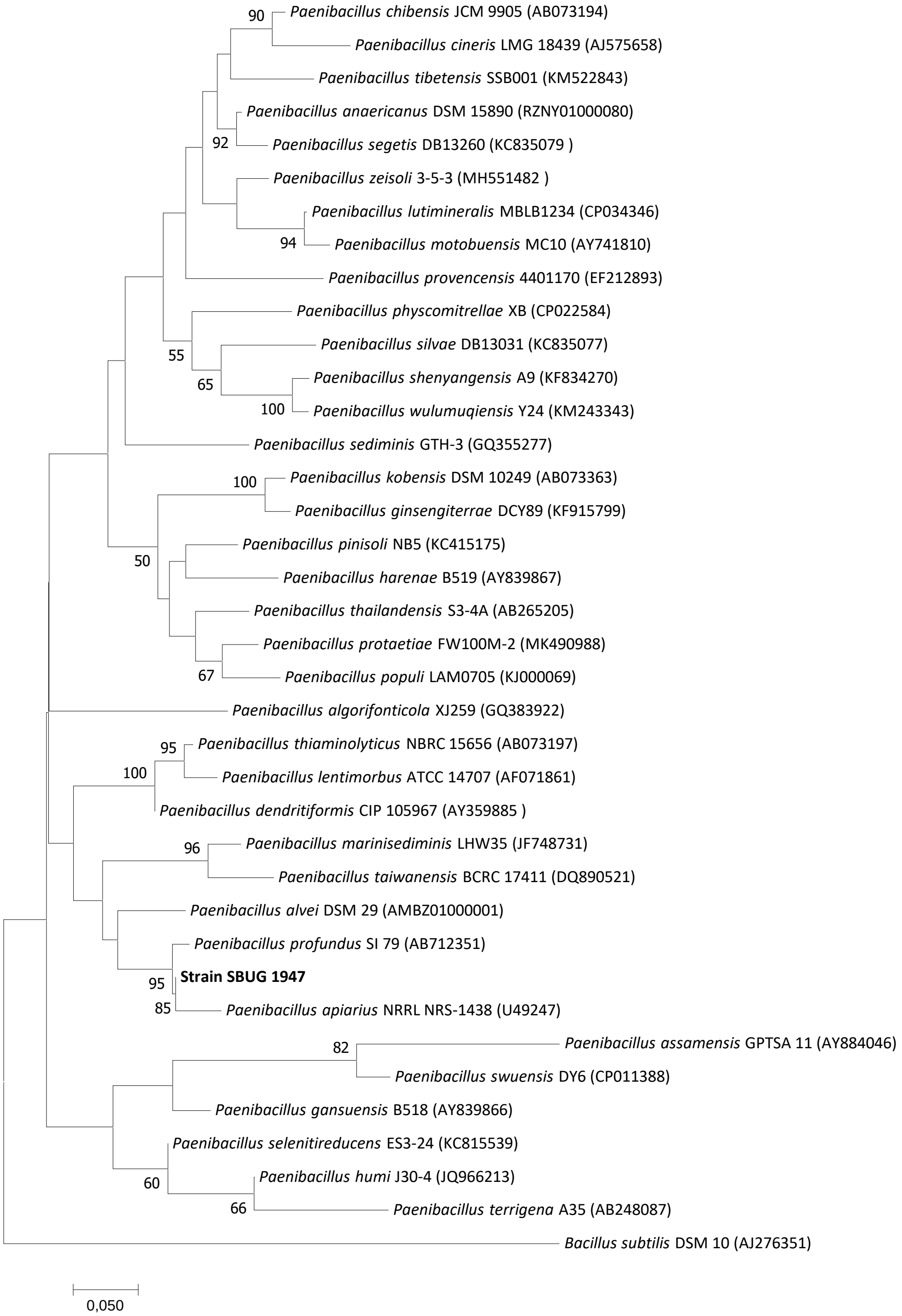

Figure S4 Phylogenetic dendrogram indicating the position of strain SBUG 1947 within the genus *Paenibacillus*. Bootstrap values ≥50% are indicated at branch points. Bar, 0.05 substitutions per nucleotide position.

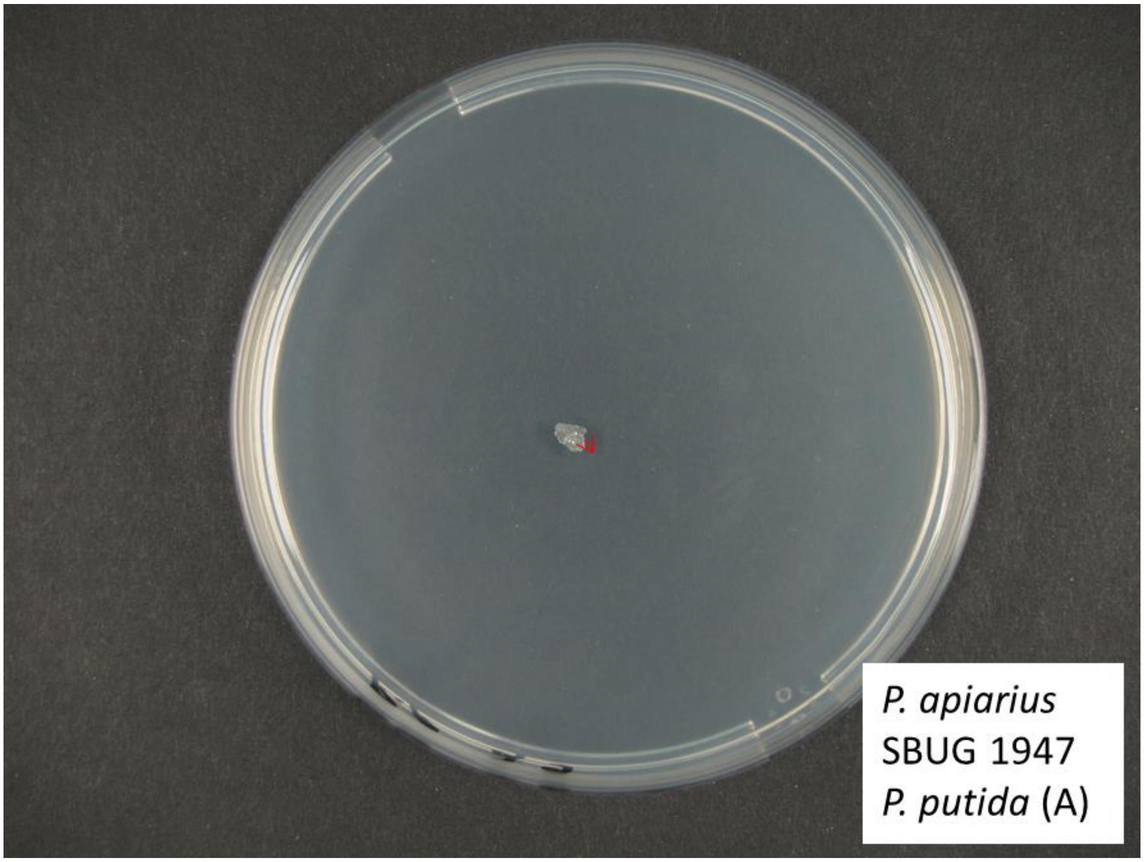

Figure S5 Image lytic radii *P. apiarius* SBUG 1947 on autoclaved cells of *P. putida*

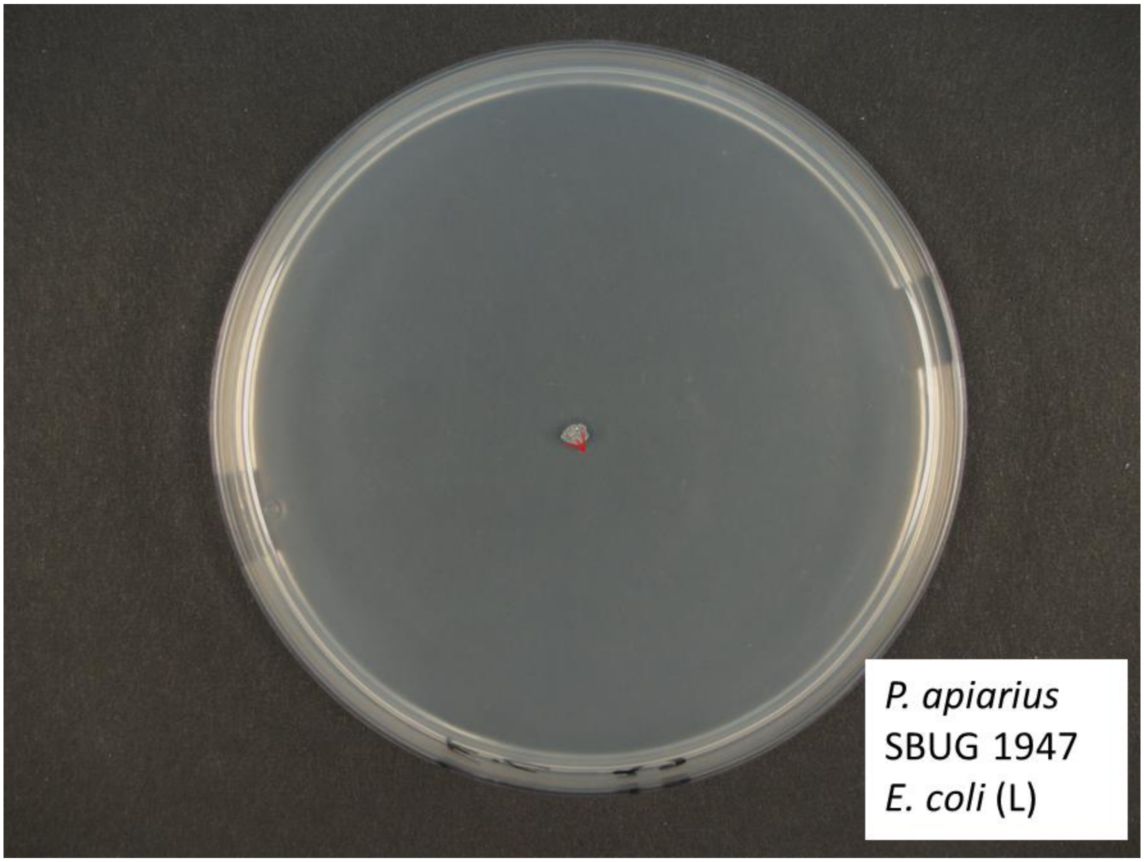

Figure S6 Image inhibition radii *P. apiarius* SBUG 1947 on living cells of *E. coli*

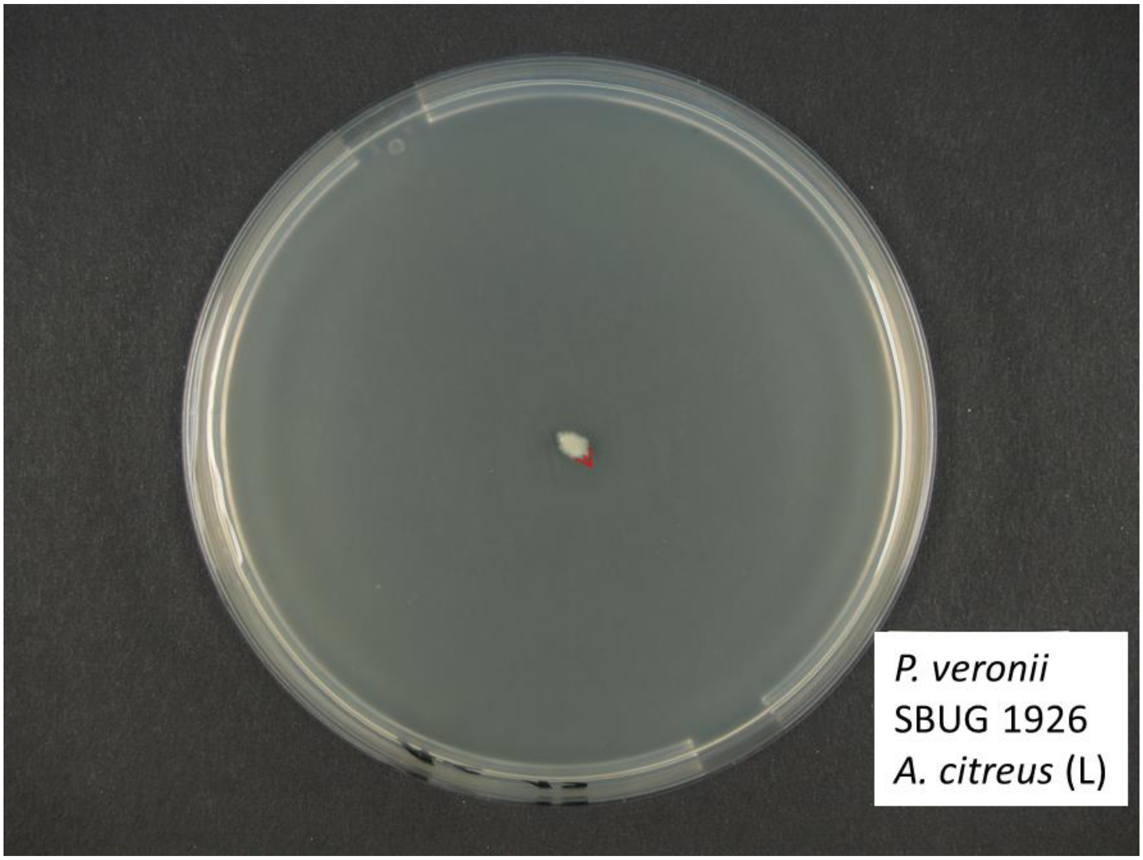

Figure S7 Image inhibition radii *P. veronii* SBUG 1926 on living cells of *A. citreus*

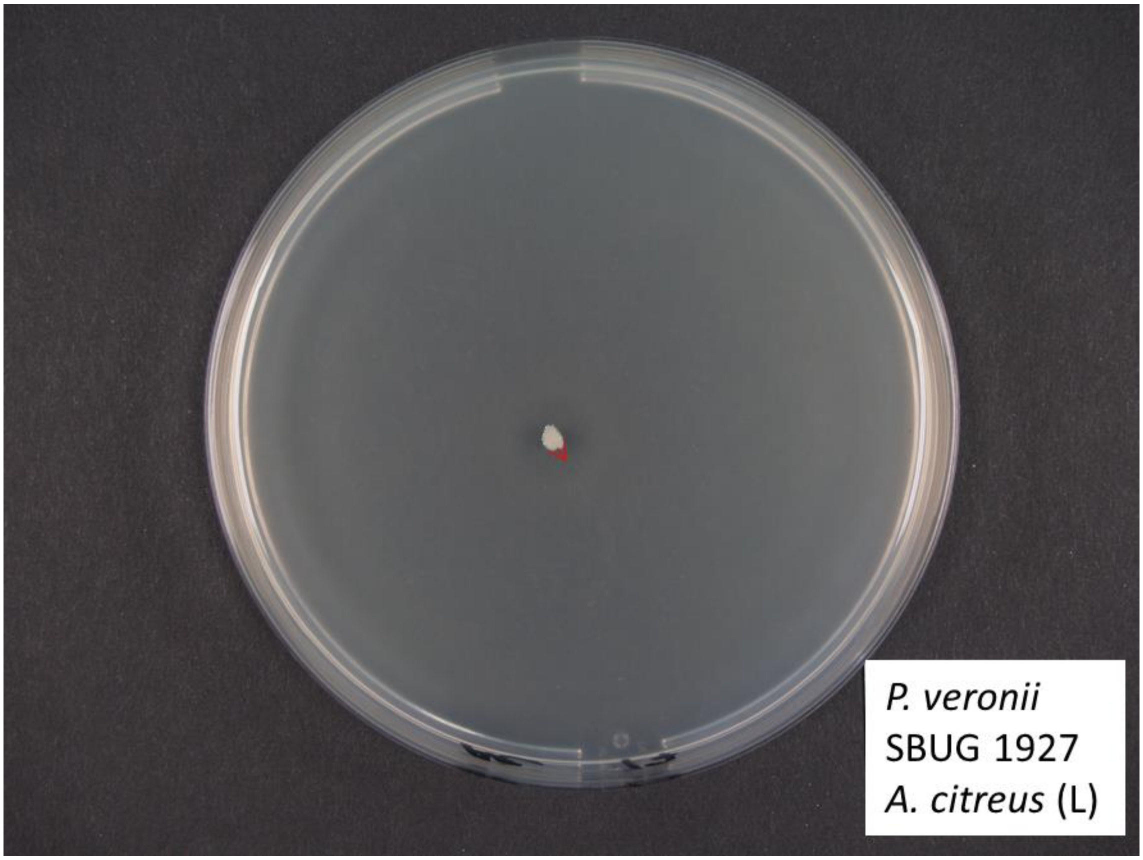

Figure S8 Image inhibition radii *P. veronii* SBUG 1927 on living cells of *A. citreus*

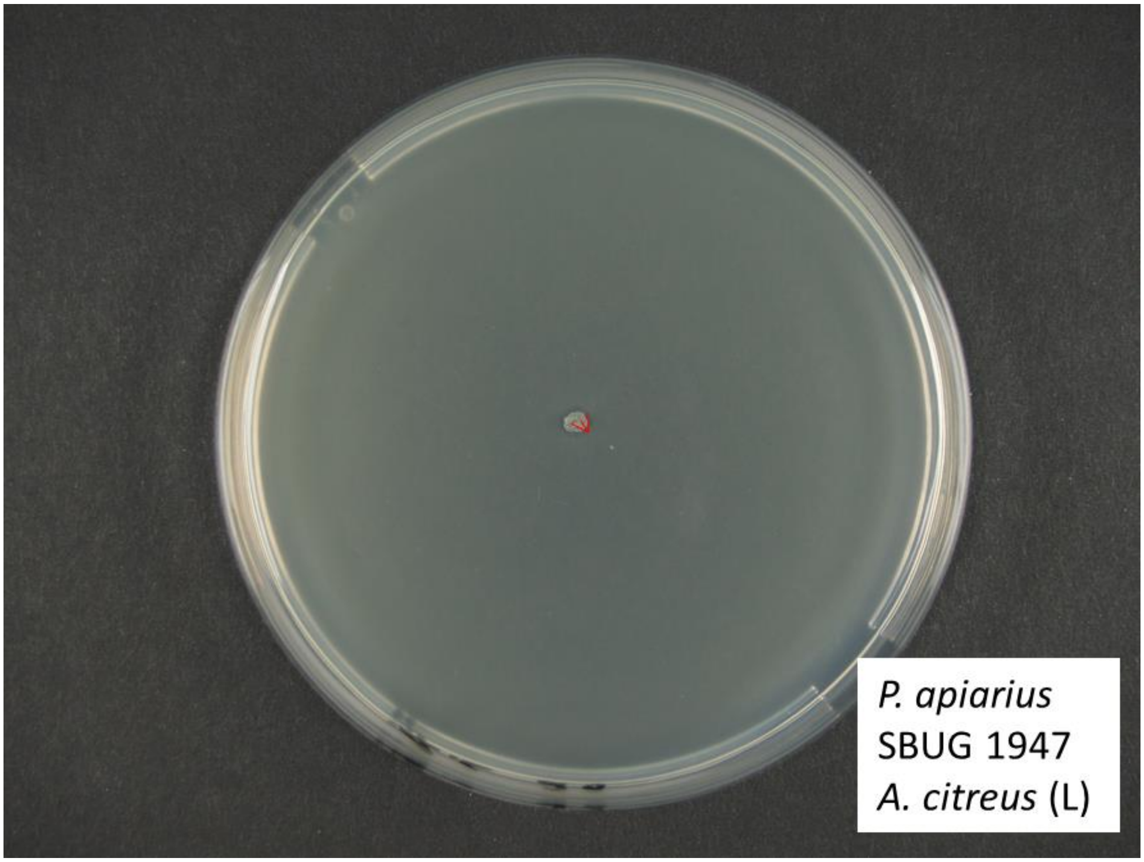

Figure S9 Image inhibition radii *P. apiarius* SBUG 1947 on living cells of *A. citreus*

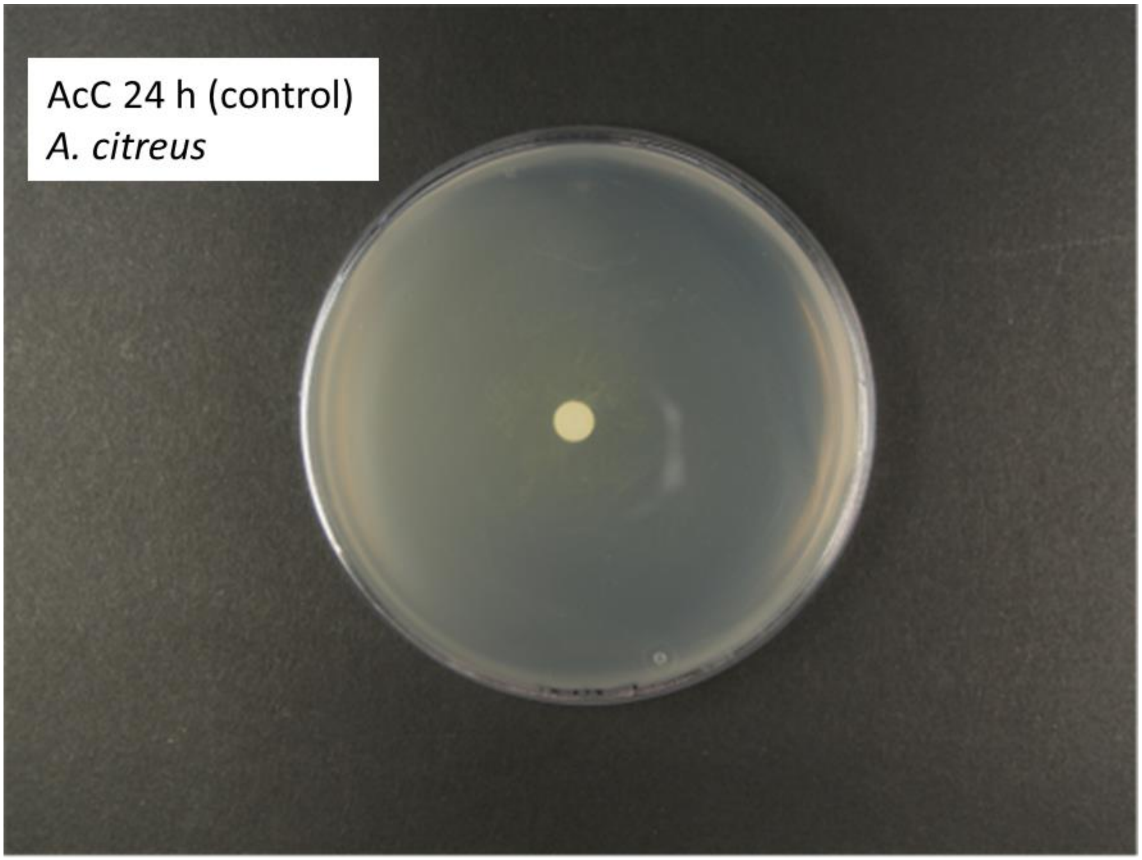

Figure S10 Image of the freeze-dried culture supernatant of *A. citreus* 24 h (negative control) on living cells of *A. citreus*

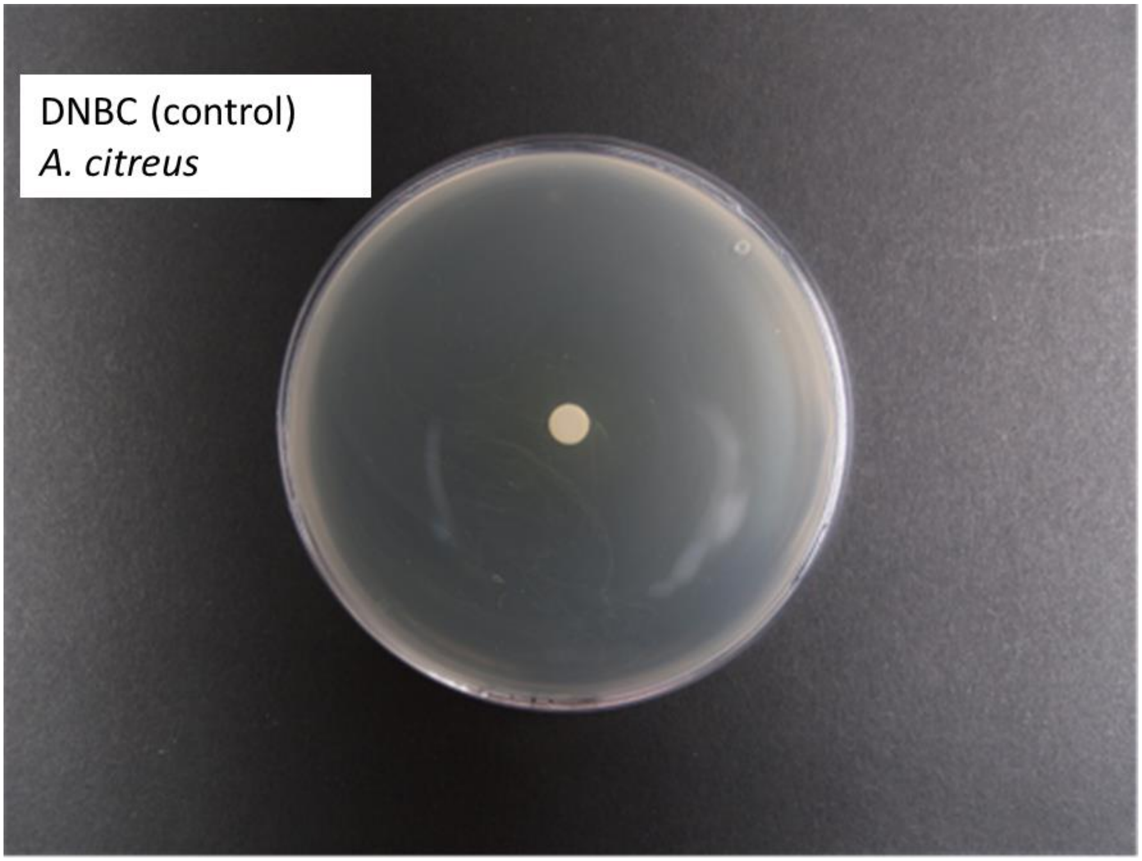

Figure S11 Image of the freeze-dried culture supernatant of diluted nutrient broth (negative control) on living cells of *A. citreus*

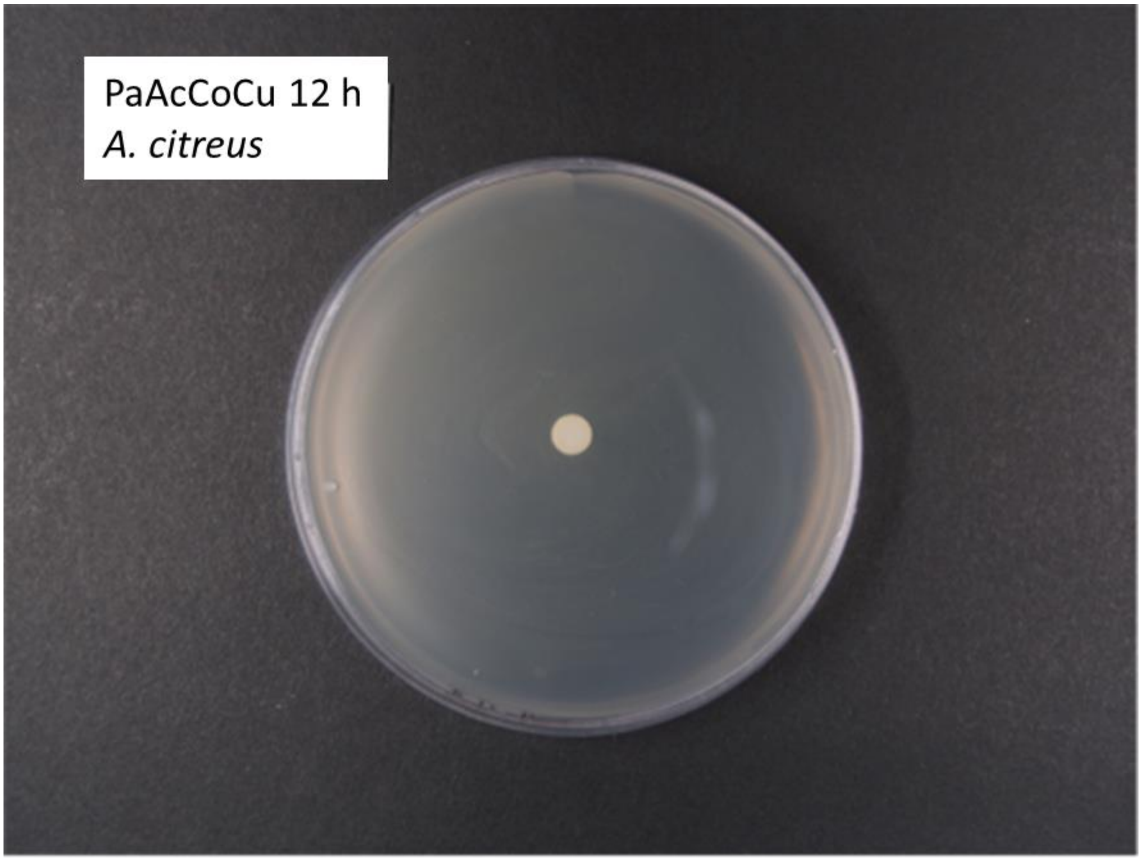

Figure S12 Image of the freeze-dried culture supernatant of *P. apiarius* and *A. citreus* co-culture 12 h (PaAcCoCu (12 h)) on living cells of *A. citreus*

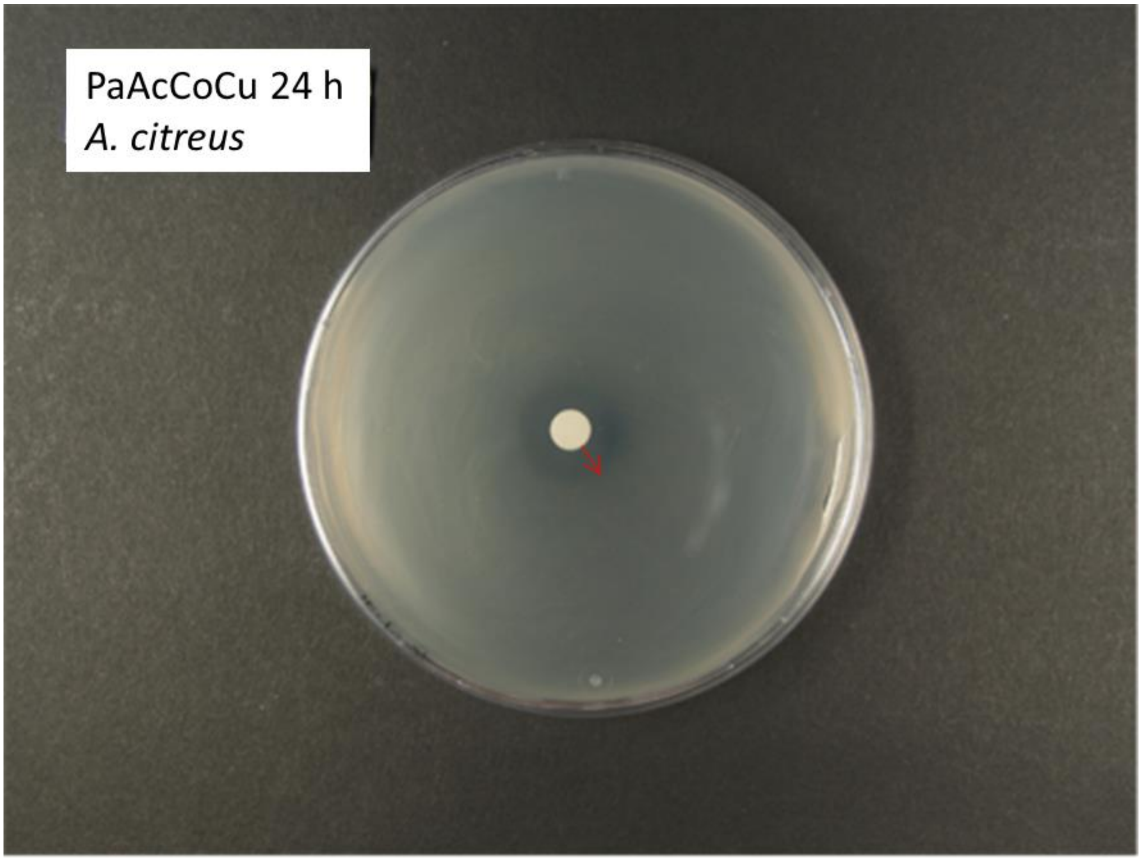

Figure S13 Image of the freeze-dried culture supernatant of *P. apiarius* and *A. citreus* co-culture 24 h (PaAcCoCu (24 h)) on living cells of *A. citreus*

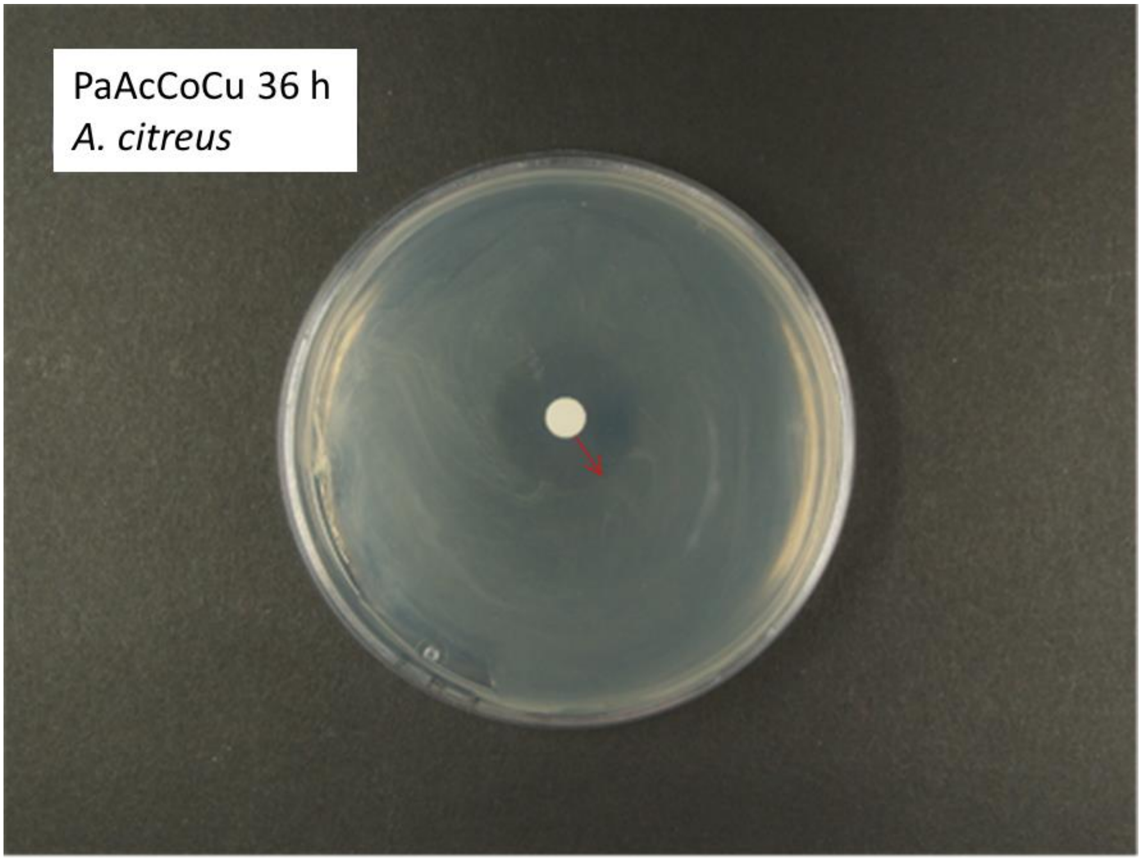

Figure S14 Image of the freeze-dried culture supernatant of *P. apiarius* and *A. citreus* co-culture 36 h (PaAcCoCu (36 h)) on living cells of *A. citreus*

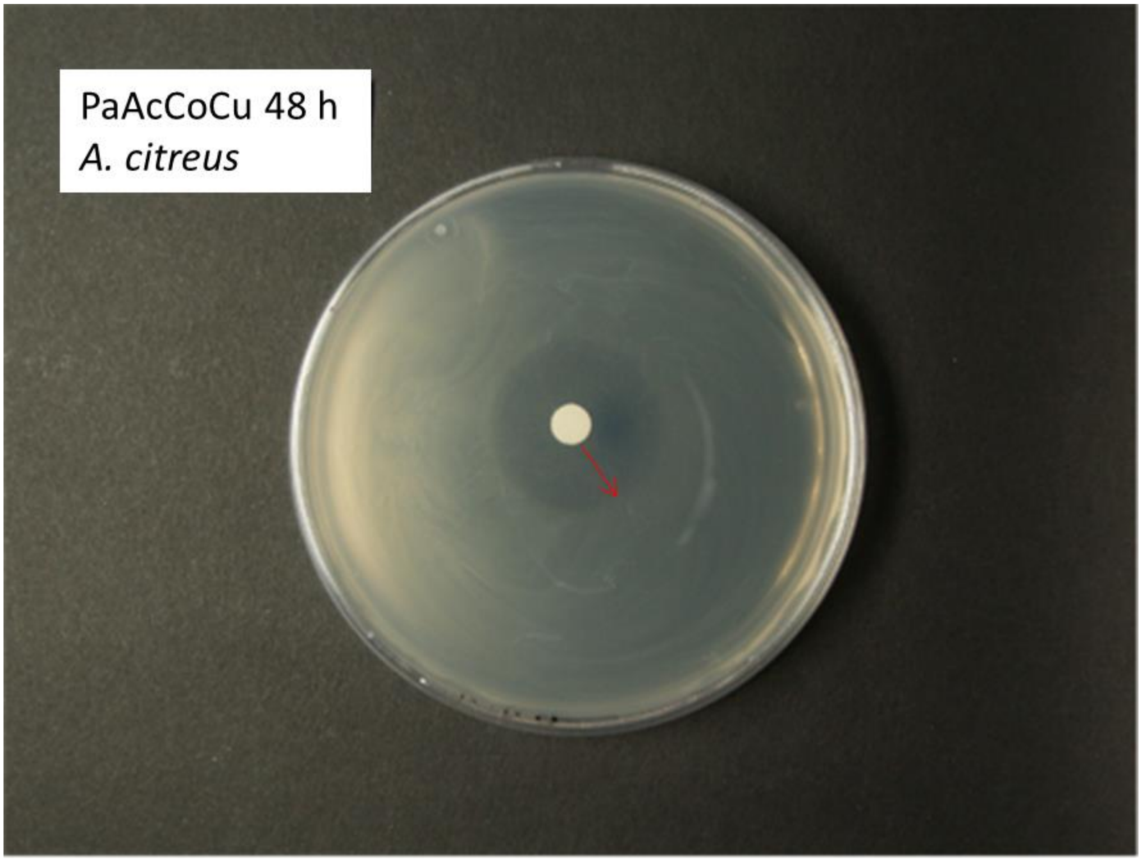

Figure S15 Image of the freeze-dried culture supernatant of *P. apiarius* and *A. citreus* co-culture 48 h (PaAcCoCu (48 h)) on living cells of *A. citreus*

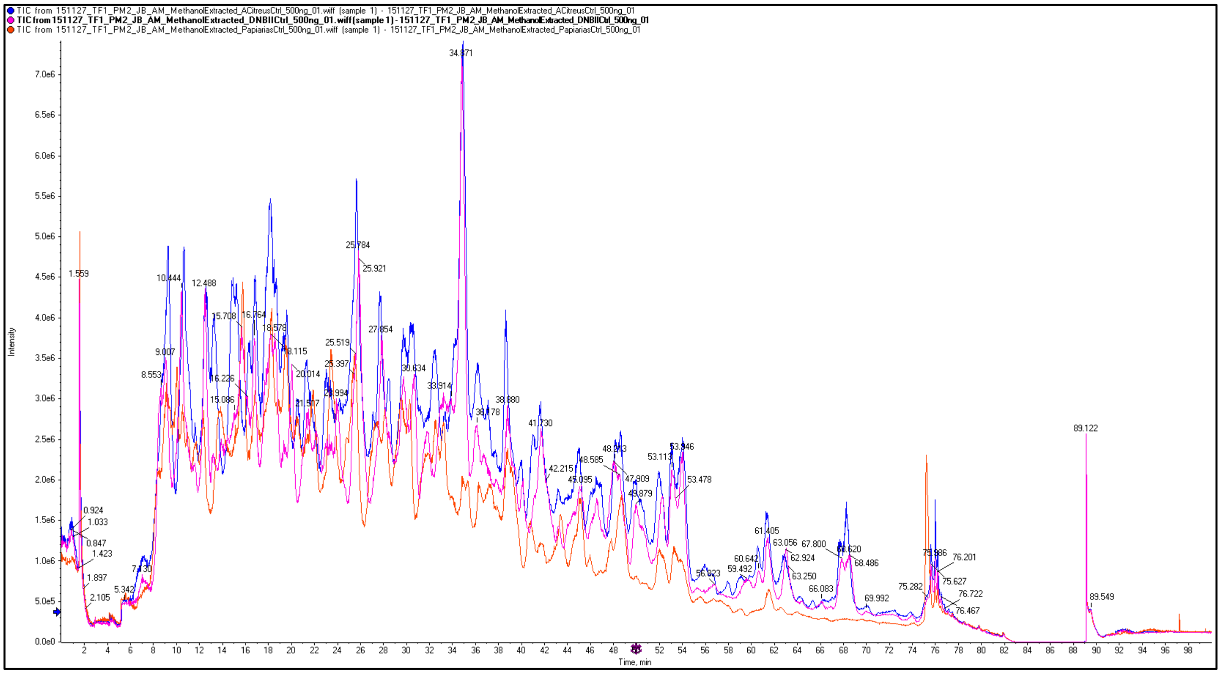

Figure S16 Liquid chromatography coupled with tandem mass spectrometry elution profiles of the culture supernatants of *A. citreus* (blue line), *P. apiarius* (red line) and the control of the culture medium (pink line).

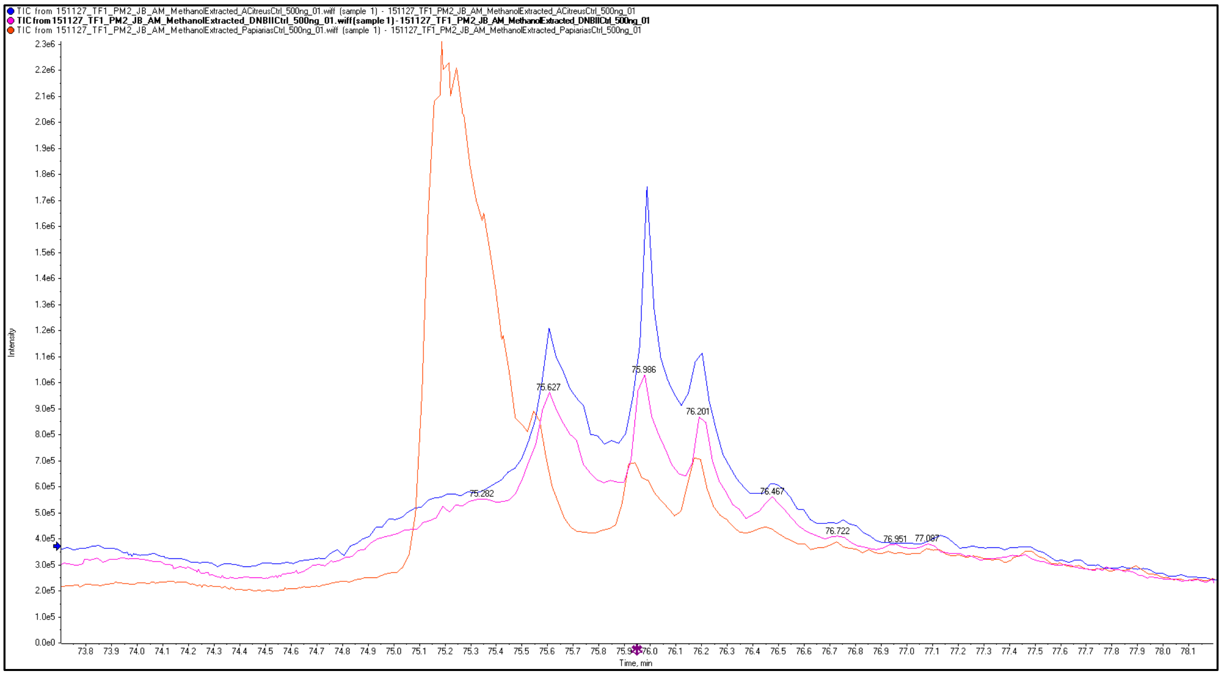

Figure S17 Detailed section of the liquid chromatography coupled with tandem mass spectrometry elution profile from Fig. S1 between 74 and 78 min, *A. citreus* (blue line), *P. apiarius* (red line) and the control of the culture medium (pink line).

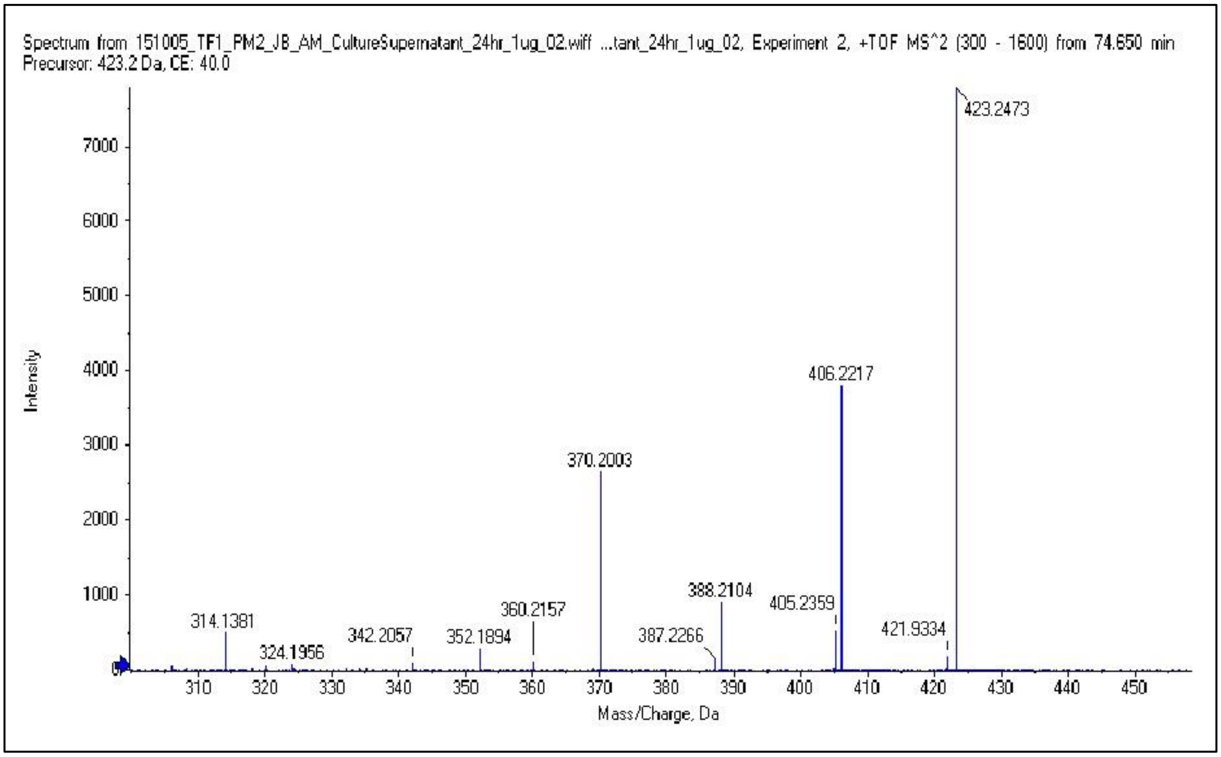

Figure S18 Mass spectrum $[M+H]^+$ of PP1 measured by tandem mass spectrometry.

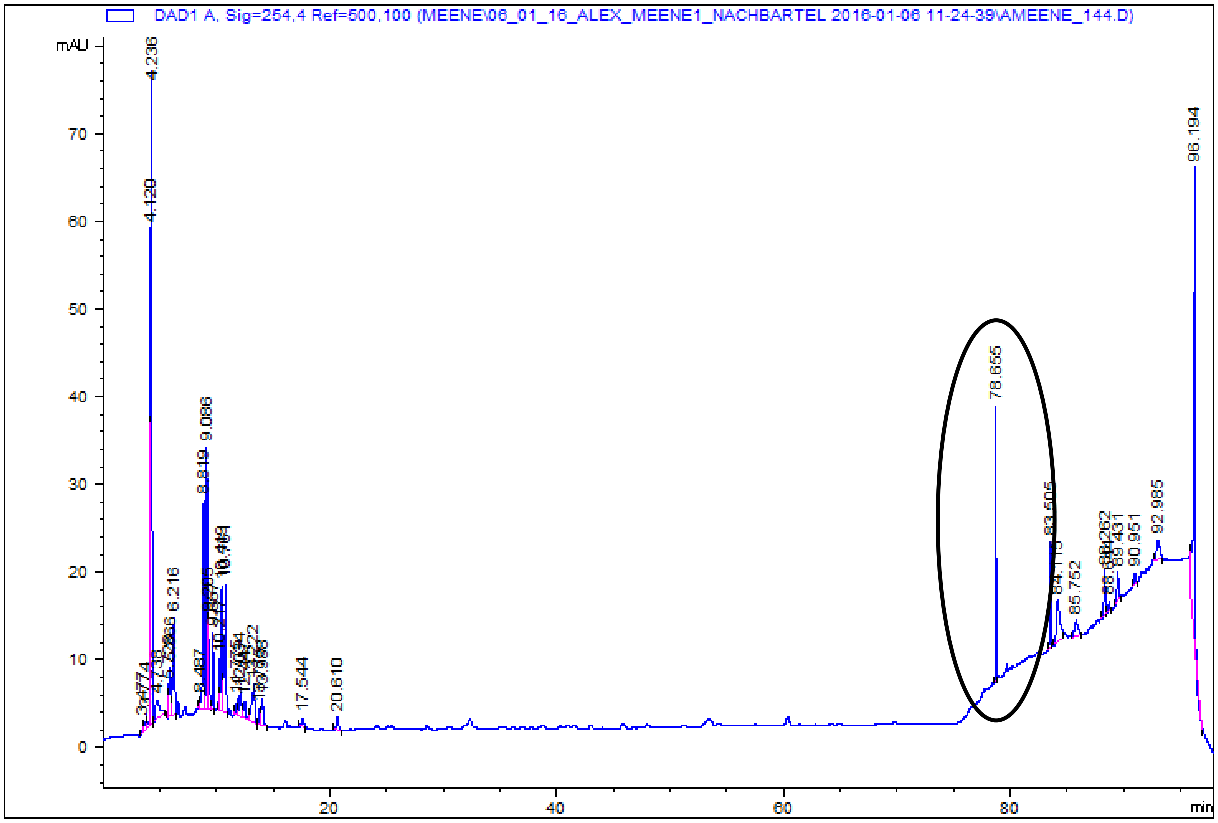

Figure S19 Liquid chromatography coupled with mass spectrometry elution profile of the co-culture supernatants of *P. apiarius* and *A. citreus*.

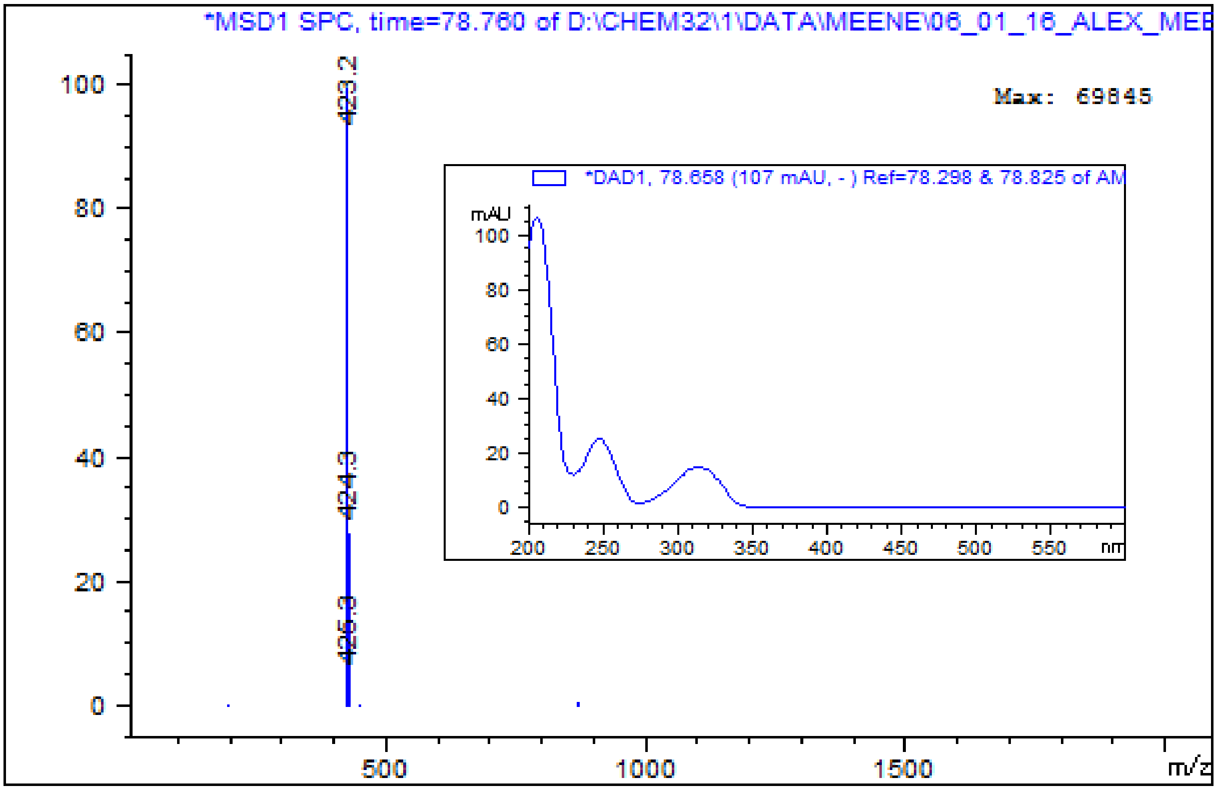

Figure S20 Mass spectrum $[M+H]^+$ and ultraviolet–visible spectrum of PP1 measured by liquid chromatography coupled with mass spectrometry and ultraviolet–visible spectroscopy.

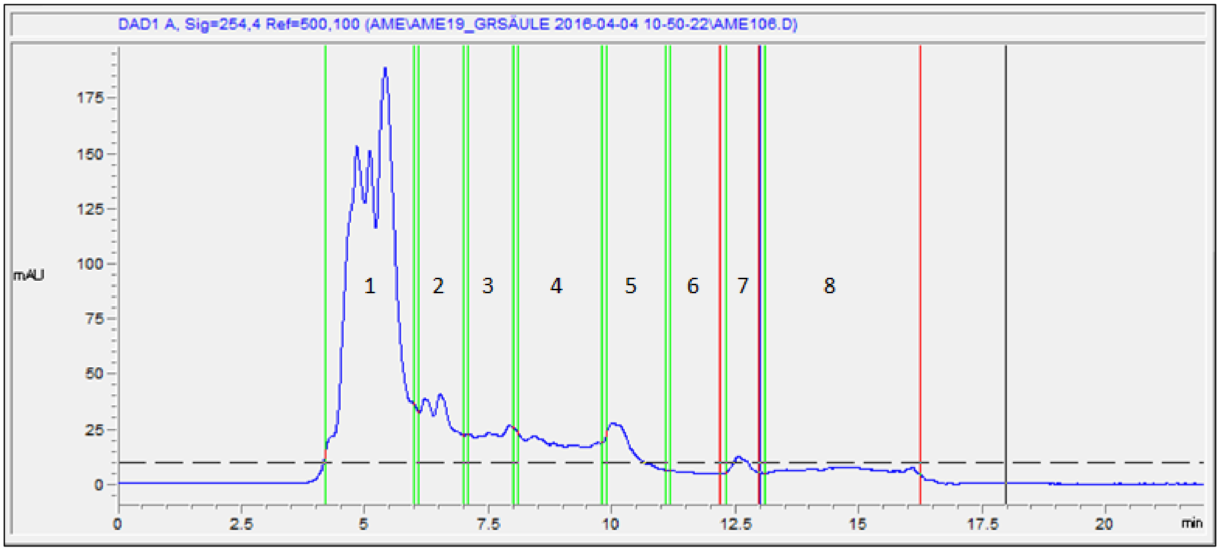

Figure S21 Fractionation scheme of preparative high-performance liquid chromatography with fractions #1 - #8 (from left to right).

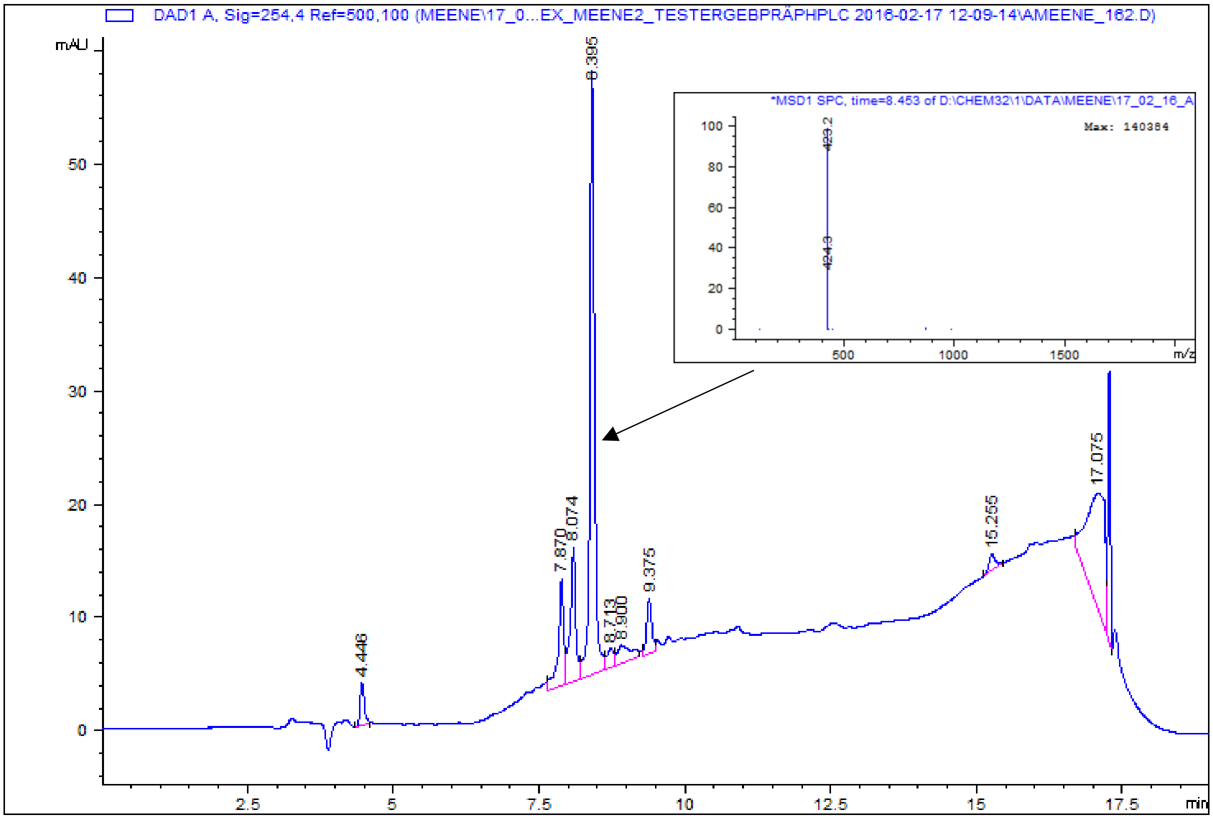

Figure S22 Liquid chromatography coupled with mass spectrometry and ultraviolet–visible spectroscopy elution profile of fraction #5 containing PP1 as main compound and mass spectrum of PP1 confirming the molecular weight of 422.2 Da.
